# Hyperbranched Polyol Process for the Synthesis of Multifunctional Cobalt Nanocomposites: Interplay of Polymer Architecture, Metal Localization and Material Properties

**DOI:** 10.3390/nano16150960

**Published:** 2026-08-04

**Authors:** Marianna P. Kutyreva, Anastasia Burmatova, Artur Khannanov, Elena Khaldeeva, Airat Kiiamov, Ruslan Batulin, Vladimir Evtugyn, Dmitry Emelianov, Liana Zubaidullina, Nikolay A. Ulakhovich

**Affiliations:** 1 A.M. Butlerov Chemical Institute, Kazan Federal University, 18 Kremlyovskaya Str., 420008 Kazan, Russia; nastyaburmatova15@gmail.com (A.B.); airatphd@gmail.com (A.K.); tokamak@yandex.ru (R.B.); vevtugyn@gmail.com (V.E.); dima_emelyan@mail.ru (D.E.); lian-usman@mail.ru (L.Z.); nikolay.ulakhovich@kpfu.ru (N.A.U.); 2 Kazan Research Institute of Epidemiology and Microbiology, 67 Bolshaya Krasnaya Str., 420015 Kazan, Russia; e_khaldeeva@mail.ru

**Keywords:** hyperbranched polyol process, cobalt nanocomposites, polyester polyols, nanoparticle morphology, nanoparticle tracking analysis (NTA), in situ FT-IR spectroscopy, magnetic properties, hemocompatibility, antimycotic activity, enzymatic activity modulation

## Abstract

A strategy based on the hyperbranched polyol process (HB-polyol process) is presented for the synthesis of hemocompatible cobalt nanocomposites Co/G_n_OH with controlled morphology and predictable functional properties. Third-generation (G_3_OH) and fourth-generation (G_4_OH) hyperbranched polyester polyols were used as smart polyol nanoreactors. We establish, for the first time, the fundamental physicochemical principles of the HB-polyol process based on a comprehensive analysis of FT-IR, UV-Vis, NMR, NTA, and TEM data. These principles encompass the stages of pre-organization, nucleation, polyol oxidation and the stabilization of cobalt-loaded metallopolymer nanocomposites within the binary [CoCl_2_–G_n_OH] system (n = 3, 4). Magnetic measurements revealed that the samples exhibit paramagnetic properties at 5 K. The size of the magnetic cores in the Co/G_3_OH samples was estimated by fitting the field-dependent magnetization curves to the Langevin function and was found to range from 1.4 nm to 7.2 nm, indicating the superparamagnetic behavior of the nanocomposites. In vitro biological tests of the Co/G_n_OH nanocomposites demonstrated high hemocompatibility, as well as pronounced modulatory and antimycotic activity across all samples. The obtained results hold promise for the development of simple design technologies for multifunctional “intelligent” materials based on metal and dendritic nanoparticles for biomedical applications.

## 1. Introduction

Currently, the development and use of technologies based on nanoscale components form the basis of technological solutions for creating materials with promising catalytic, magnetic, mechanical, optical, electrical, and biological properties [1,2,3,4,5]. These include the creation of complex multicompound nanoscale objects, which enable the creation of polyfunctional or “smart” materials. The effectiveness of these materials is provided by the combination of their chemical composition and their dimensional effects. To develop technological solutions, it is necessary to solve several key tasks. The first task is the development of simple methods for the synthesis of nanocomposite or hybrid particles. The second aspect is the identification of the conditions and components of the system within the framework that effectively controls the morphology and, consequently, the functional activity of complex nanoparticles.

An analysis of the latest developments in nanotechnology highlights the scientific and practical significance of the “polyol process” as a highly efficient, affordable and zero-waste technology for producing complex metal-loaded composite nanomaterials.

This approach has several advantages, including accessibility, versatility, and scalability. It allows for precise control over the shape and size of synthesized nanoparticles [6,7,8]. Only two components serve as the main reactants: a metal nanophase precursor compound and a polyol, which simultaneously acts as a reducing agent and stabilizer for the metal nanoparticles. The metal containing precursor compound serves as a starting point for the metallic nanophase while the polyol acts as a reducing agent and stabilizer. By adjusting the synthesis conditions such as the nature and concentration of these components, as well as the temperature and pH of the reaction medium, it is possible to precisely control the composition, structure, dimensions and morphology of the resulting metal particles. Scientific publications and monographs contain a sufficient number of examples demonstrating the applicability of this polyol process in the production of noble metal nanoparticles [9,10]. The use of polyol processes for the production of magnetic nanoparticles from transition metals (Fe, Co, etc.), as well as their compounds, has been initiated relatively recently. An analysis of current information allows us to make a number of generalizations and identify a number of unresolved problems in this area.

In situ synthesis of magnetic nanoparticles (MNPs) using low-molecular-weight polyhydric alcohols (ethylene glycol, propylene glycol, glycerin, etc.) as polyols is the most studied method [8,9]. It has been theoretically and experimentally shown that salts of strong acids (chlorides, nitrates and sulfates) cannot be used in a two-component system with a linear diol to produce metal nanoparticles. A solution is proposed in the work by J. Balachandran et al. [11] by neutralizing the effect of the pKa through the addition of NaOH to the reaction system while maintaining the ionization of the polyol. This means that using alkali as the ionizing component will make the synthesis invariant with respect to the anion of the precursor salt.

A key challenge in implementing the “low-molecular-weight” polyol process is controlling the morphology of magneto-active nanoparticles. Solving this problem entails introducing additional stabilizing agents into the polyol process. Long-chain alkylamines (oleylamine, etc.) [12,13], polymers (polyvinylpyrrolidone—PVP, Polyethylene glycol—PEG, etc.) [14,15] and simple anions (carboxylate and halide anions) [16,17] are most frequently used for this purpose. Polyvinylpyrrolidone and polyethylene glycol of various molecular weights are most frequently used to control the morphology of magnetic nanoparticles. The work by [15] presents the polyol synthesis of cobalt nanoparticles, which was carried out by boiling a mixture of cobalt acetate, diethylene glycol (DEG), water and PVP at 200 °C. It was found that the addition of water (water/DEG = 0.017:1) and polyvinylpyrrolidone to the reaction mixture makes it possible to obtain spherical Co^0^ nanoparticles, whereas non-spherical particles are obtained in the absence of these components. Using nickel nanoparticles as an example, the effect of the molecular weight of polyethylene glycol on the shape and size of nanoparticles was studied [16]. It was shown that an increase in the molecular weight from 200 g/mol to 6000 g/mol leads to an increase in the proportion of snowflake-like nanoparticles and a decrease in the dimensional characteristics of nanoparticles from 150 nm to 75 nm.

The transition to the use of high-molecular-weight linear polyols, such as polyvinyl alcohol [18] and polyethylene glycol [19,20,21,22], within the polyol method for nanoparticle synthesis enables the optimization of the reagent composition. Polyethylene glycol (PEG) of various molecular weights is the most extensively studied polymer for this synthesis method [23,24]. Despite the limited information available in this area, it has been conclusively demonstrated that the nucleation process does not require additional stabilizing agents and is controlled by the number of OH groups in the polyol, the M^n+^/OH_polyol_ ratio and the molecular weight of the linear polyol [8,9,19,23,25]. Using the synthesis of iron nanoparticles in a PEG200 medium as an example, it was demonstrated that increasing the Fe^3+^/PEG200 ratio leads to a reduction in nanoparticle size [19]. Conversely, increasing the molecular weight of the linear polyol results in a lower nucleation rate and larger crystal sizes [8,9,25]. Our systematic studies have established the limit of the reducing activity of PEG—4000 in the synthesis of cobalt nanoparticles at an Co^2+^/OHPEG ratio of at least 1:10. At the same time, the Co/PEG—4000 nanosystem stabilizes only after 14 days and remains stable for no more than 21 days [24].

Thus, the search for new methods of polyol synthesis for nanoparticles—including the identification of new polyols as the primary components of this process—represents an important fundamental and practical challenge in the field of nanomaterial chemistry.

To advance this area and overcome the aforementioned challenges, we propose a nature-inspired polyol synthesis technology based on the use of dendrite-like hyperbranched polyols (HB-polyols) with a 3D architecture as multifunctional nanoreactors for the polyol process. HB-polyols have a dendrite-like architecture and are nanoscale macromolecules [26,27]; they possess a high number of hydroxyl groups located both in the shell and core of the macromolecule, greater branch mobility than dendrimers and therefore higher chemical activity, and exhibit aggregation activity. According to their properties, HB-polyols can simultaneously be positioned as effective reagents of polyol synthesis, as adaptive stabilizers of the metallic nanophase [28,29,30] and as nanoscale components of the target metal polymer composite. Previously, we have shown the possibilities of implementing a hyperbranched polyol process (HB-polyol process) for the synthesis of nanoscale cobalt composites in a medium of hyperbranched fourth-generation polyester polyol, **G_4_OH** [31]. It has been established that HB-polyol can act both as a reducing agent and as a reservoir stabilizer of cobalt nanoparticles (by the type of stabilization in the “dendritic window”). However, this work is primarily limited to demonstrating the reducing capability of the **G_4_OH** polyol. In the present manuscript, we conduct, for the first time, a systematic comparison of the HB-polyol process using polyols of different generations (**G_3_OH** and **G_4_OH**) and establish a fundamental relationship between the dendrimer-like polymer architecture, the localization of the metal ion, the extent of its oxidation, and the chemical composition of the polymer’s oxidized surface shell during stabilization.

## 2. Materials and Methods

### 2.1. Materials

For the synthesis of cobalt-containing nanocomposites (**Co/G_n_OH**), hyperbranched bis-MPA polyester polyols, generation 3—**G_3_OH** (Sigma-Aldrich, St. Louis, MO, USA, CAS: 326794-48-3, 32 hydroxyl groups, M_r_ = 3607 g·mol^−1^, hydroxyl number 500 mg KOH × g^−1^), generation 4—**G_4_OH** (Sigma-Aldrich, St. Louis, MO, USA, CAS: 326794-48-3, 64 hydroxyl groups, M_r_ = 7323.32 g·mol^−1^, hydroxyl number 486 mg KOH × g^−1^) and salt CoCl_2_ × 6H_2_O (99%, Alfa Aesar, Haverhill, MA, USA) as a cobalt precursor were used.

To study the hemolytic activity of **Co/G_n_OH**, blood from healthy donors was obtained from the blood donor center in Kazan and anticoagulated with 3% sodium citrate.

The evaluation of proteolytic activity in the presence of **Co/G_n_OH** was carried out for the enzyme–substrate system: chymosin (CHY-MAX Powder Extra NB, Hansen, CAS 9001-98-3 Rennet Powder 20,000 u/g)–bovine hemoglobin (SKU: 212392, BBL^TM^, Franklin Lakes, NJ, USA). Chymosin and hemoglobin solutions were prepared in PBS at pH 3.58, stored at 4–5 °C and used within 24 h.

The antimycotic activity of **Co/G_n_OH** was evaluated by disk diffusion tests on cell cultures of *Candida albicans*, *Cryptococcus laurentii*, *Aspergillus fumigatus* and *Aspergillus niger* provided by the Laboratory of Fungal Allergens of the Kazan Research Institute of Epidemiology and Microbiology of the Russian Federation.

Acetone, DMSO, and petroleum ether purified according to standard procedures were used as solvents for the synthesis and isolation of cobalt-containing nanocomposites. **Co/G_n_OH** samples for TEM were prepared in methanol. In experiments to study the properties of **Co/G_n_OH,** we used deionized water (Ω = 18.2 MΩ × cm at 25 °C, χ = 0.055 µS/cm, particle number of 0.22 µm/mL < 1) and phosphate-buffered saline (PBS) with pH = 3.58.

### 2.2. Equipment

The FT-IR spectra were recorded on a Spectrum 400 (PerkinElmer, Waltham, MA, USA) ATR-FT-IR spectrometer. The FT-IR spectra from 4000 to 400 cm^−1^ were considered in this analysis. The spectra were measured with 1 cm^−1^ resolution and a 64 scans co-addition.

To establish the mechanism of oxidation of polyester polyol **G_n_OH**, FT-IR spectra were recorded on a Spectrum 400 Fourier spectrometer (PerkinElmer, Waltham, MA, USA) with a diffuse reflectance attachment with a compact temperature controller from “PIKE technologies” in the temperature range 35–210 °C and 4000–400 cm^−1^ (1 cm^−1^ resolution, 16-scan accumulation, shooting range 4000–400 cm^−1^).

The electronic absorption (UV-Vis) spectra were recorded on a Lambda 750 (PerkinElmer) in the wavelength range of 200–1000 nm at T = 25 ± 0.01 °C, using a temperature-maintaining system including a cell holder flow thermostat, “Julabo MB-5A”, and a Peltier PTP-1 thermostat (PerkinElmer, Waltham, MA, USA). Quartz cells with a thickness of 1 cm were used for the measurements. The measurement accuracy for absorbance (A) was ±1%.

The **Co/G_n_OH** particle characteristics in the solution were studied by the Nanoparticle Tracking Analysis (NTA) method on a NanoSight LM-10 instrument (Malvern Panalytical, Malvern, UK). The CMOS camera C11440-50B with an image capture sensor FL-280 from Hamamatsu Photonics (Hamamatsu, Japan) was used as a detector. The measurements were carried out in a special cuvette for aqueous solutions, equipped with a laser with a wavelength of 405 nm (CD version S/N 2990491); the O-ring was made of Kalrez material. The temperature in the chamber was determined using an OMEGA HH804 contact thermometer (Engineering Inc., Norwalk, CT, USA) for all measurements. For the spectra fitting, the OriginPro 2024 (SR1 10.1.0.178) program package was used; the Gauss function was used throughout.

Thermogravimetric analysis (TG) was performed using an STA449 F1 Jupiter thermal analyzer (Netzsch, Selb, Germany) with a temperature rate of 10 K × min^−1^ in an argon atmosphere and a total flow rate of 75 mL·min-1. The analysis was performed in a temperature range of 40–600 °C in an Al crucible with a volume of 40 μL that had lids with 3 holes, each 0.5 mm in diameter. Experiments on the thermal stability (DTG) of the composites were performed on a TG209 F1 Libra precision thermobalance (Netzsch GmbH, Selb, Germany) in a synthetic air atmosphere. For the experiment, a sample of colloidally stable particles was precipitated with water and washed successively with acetone and diethyl ether.

The phase composition of **Co/G_n_OH** particles was established by powder X-ray diffraction (XRD), which was acquired with a Bruker D8 Advance using Cu Kα irradiation (λ = 1.5418 Å) in the Bragg–Brentano geometry: the scan rate was 0.18°/min; the 2θ angle range was from 7° to 100°; and the step was 0.015°. For the experiment, a sample of colloidally stable particles was precipitated with water and washed successively with acetone and diethyl ether.

The particle morphology of **Co/G_n_OH** particles was established by transmission electron microscope (TEM) imaging, which was carried out using the Hitachi HT7700 transmission electron microscope (Tokyo, Japan) at an accelerating voltage of 100 kV in TEM mode. The size and shape of the synthesized nanoparticles were estimated using ImageJ (version 1.54g). The chemical composition of the metal phase was determined by the EDX method.

Ultrasonic treatment of dispersions for sorption studies was carried out in a Sapphire ultrasonic bath (T = 25 °C, operating frequency 35 kHz).

The magnetic properties of iron nanoparticles were measured using a PPMS-9 (Quantum Design, San Diego, CA, USA) equipped with a vibration sample magnetometer (VSM). Zero-field-cooling (ZFC) and field-cooling (FC) measurements were performed in a 20 Oe magnetic field over the temperature range 7–300 K. The field dependences of the magnetization were measured at 7 K in the magnetic field range from −1 T to 1 T.

### 2.3. Synthesis of Colloidal Cobalt-Containing Nanocomposite Co/G_n_OH by Hyperbranched Polyol Process

The main approach to the synthesis of nanocomposites by the hyperbranched polyol process is presented in the article [31]. Polyester polyol **G_n_OH** (3 g) was dissolved in 4.75 mL of DMSO and was mixed at room temperature until the polymer was completely dissolved. Then, CoCl_2_ × 6H_2_O (0.0127 g, 0.0254 g, 0.0424 g, and 0.0636 g for **G_3_OH**; 0.0124 g, 0.0247 g, 0.0412 g, and 0.0618 g for **G_4_OH**) was added, and the solution was heated at 40 °C until the salt was completely dissolved. Molar ratios of M^n+^/OH_polyol_ were ν_Co(II)_/ν_OHpolyol_ = 1:500 for **Co/G_3_OH-1** and **Co/G_4_OH-1**, 1:250 for **Co/G_3_OH-2** and **Co/G_4_OH-2**, 1:150 for **Co/G_3_OH-3** and **Co/G_4_OH-3** and 1:100 for **Co/G_3_OH-4** and **Co/G_4_OH-4**. The reaction mixture was left at a stable temperature for 1 day for self-organization and then was heated to 240 °C in steps of 10 °C with constant stirring. Solid NaOH was added to all samples (molar ratio of ν_NaOH_/ν_Co(II)_ = 1:10) before the heating stage over a period of 8 h. The formation of a colloidal stable cobalt-containing nanocomposite **Co/G_n_OH** was accompanied by a change in the color of the reaction mixture from blue to green.

### 2.4. Sorption Studies

The sorption of Co^2+^ ions by the polyester polyol **G_n_OH** matrix was carried out under static conditions in DMSO. A series of solutions was prepared with a constant concentration of polyol (0.125 g·mL^−1^) and a variable concentration of CoCl_2_⋅6H_2_O (0.05–0.8 mol·L^−1^) in DMSO. Then, mixtures were treated with ultrasound for 1 h and incubated at room temperature for 24 h. The resulting high-molecular associates [Co^2+^-**G_3_OH**] and [Co^2+^-**G_4_OH**] were separated by centrifugation (v = 10,000 rpm, t = 5 min) and the free concentration of the Co^2+^ ion in the solution was determined by UV–Vis spectrophotometry according to the calibration graph at λ = 530 nm:

*A*_530_ = (12.06 ± 0.42) × C*_Co_*_(*II*)_ − (0.15 ± 0.10) *R* = 0.9979

Intrinsic absorption of **G_n_OH** was taken into account in the baseline. In parallel, control experiments were carried out with CoCl_2_ × 6H_2_O solutions at the same concentrations but without polymers. The sorption isotherm was plotted in the coordinates c^S^_Co2+_ from c^0^_Co2+_, where c^S^_Co2+_ is the sorbed concentration of Co^2+^ and c^0^_Co2+_ is the initial concentration of Co^2+^ in the solution.

### 2.5. Hemolysis Assay

The hemolysis assay was performed according to the method in [32]. Blood erythrocytes were separated from the plasma and leukocytes by centrifugation (5000× *g*, 5 min) at 4 °C and washed three times with phosphate-buffered saline (PBS). They were used immediately after isolation. To study the effects of the nanocomposites on hemolysis, the red blood cells (RBCs) were suspended in polymer solutions in PBS at a hematocrit of 1% and incubated for 0.5 h at a temperature of 20 °C. The incubated suspensions were centrifuged at 1000× *g* for 5 min. For reference, the RBCs were treated with double-distilled water, which causes 100% hemolysis. The hemolysis coefficient (HC, %) was determined by releasing hemoglobin in the supernatants and was measured spectrophotometrically by absorbance at 540 nm:

$$HC\left[\%\right]={\frac{A-A_{-}}{A_{+}-A}}_{-}\times 100\%,$$

where *A* is the optical density of the RBCs incubated with nanocomposites, *A_−_* is the optical density of the sample in PBS, and *A_+_* is the optical density of the RBCs in water (100% hemolysis). The nanocomposites themselves contributed no more than 0.1% of the absorbance at 540 nm. The results are expressed as mean ± standard deviation, n = 5. The value of HC_50_ corresponds to the concentration of **Co/G_n_OH** at which 50% hemolysis of erythrocytes is observed.

### 2.6. Enzymatic Activity Studies

The properties of **Co/G_n_OH** as a synthetic modulator of enzymatic activity were evaluated using the chymosin *(enzyme)*–hemoglobin *(substrate)* system by UV-Vis spectrophotometry. To determine the proteinase activity, solutions of chymosin (2 mg·mL^−1^) and hemoglobin (Hb) (2 mg·mL^−1^) in PBS medium (pH = 3.58) were prepared. Then, 0.1 mL of chymosin was added to a mixture containing 0.5 mL of Hb with and without **Co/G_n_OH** (0.001–10 mg·mL^−1^). The total volume of the sample was 1 mL, the chymosin concentration in the sample was 0.2 mg·ml^−1^, and the hemoglobin concentration in the sample was 1 mg·ml^−1^. After mixing and incubation at T = 50.0 ± 0.01 °C for 30 min, the samples were centrifuged at 10,000 rpm for 20 min. The supernatant was transferred into a quartz spectrophotometric cuvette and the electronic absorption spectrum was recorded in the wavelength range of 250–500 nm.

The concentration of Hb (c_Hb_ mg·mL^−1^) after proteolysis was estimated according to the calibration graph as follows:

*A*_280_ = (−0.0172 ± 0.0146) + (0.0017 ± 0.0002) × *c_Hb_*, R = 0.9996

The contribution of enzyme absorption was taken into account as a baseline. Each experiment was repeated at least three times.

The activity of chymosin (A_E_, mg·mL^−1^·min^−1^) was determined by the change in the rate of the catalytic reaction compared to the non-catalytic one. The reaction rate was indicated as the change in the substrate concentration (mg × mL^−1^) per unit of time (min):

$$A_{E}\left[mg\times {mL}^{-1}\times {min}^{-1}\right]=\frac{C_{Hb}^{0}-C_{Hb}}{t},$$

where $C_{Hb}^{0}$ is the initial concentration of hemoglobin and $C_{Hb}$ is the concentration of the hemoglobin solution after proteolysis.

Dependences, *A_E_ = f(lgC*_Co/GnOH_*)*, were built according to the experimental data. Chymosin activity in the absence of the cobalt-containing nanocomposite **Co/G_n_OH** was taken as *A_E_*^0^.

### 2.7. Antimycotic Study

The antimycotic activity of the cobalt-containing nanocomposite **Co/G_n_OH** was evaluated at the Kazan Research Institute of Epidemiology and Microbiology on cell cultures of *Candida albicans*, *Cryptococcus laurentii*, *Aspergillus fumigatus* and *Aspergillus niger.* Studies of the antimycotic activity of **Co/G_n_OH** were carried out using the disk diffusion test on modified Saburo agar. Plants of test cultures (spore suspension) were applied at the rate of 1 million CFU/cup. Solutions of test substances were applied to sterile paper disks, dried until the solvent was completely removed, placed in the cup with the culture, and incubated for 2–4 days at 28 °C. To control fouling, cultures with disks were incubated for up to 7 days.

## 3. Results and Discussion

**Figure 1:**
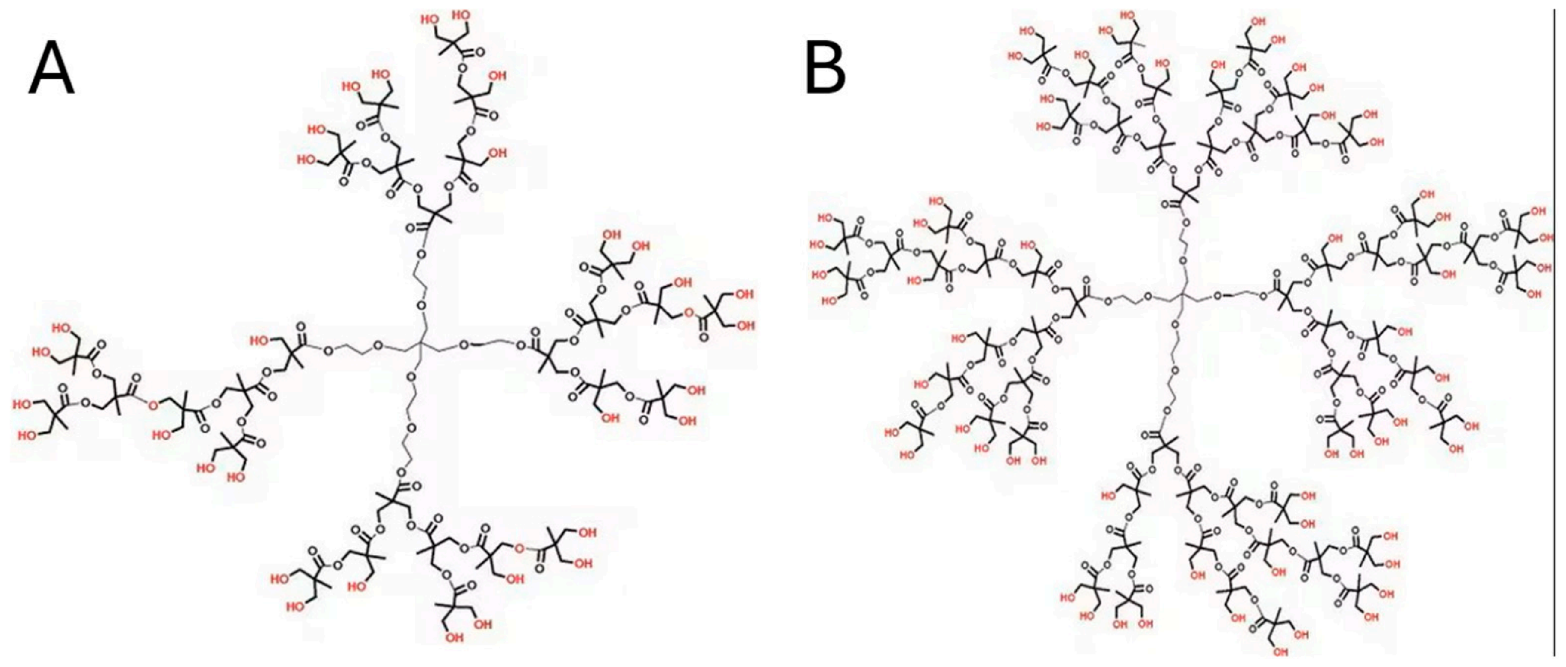
Structures of hyperbranched polyester polyols **G_3_OH** (**A**) and **G_4_OH** (**B**).

The hyperbranched polyester polyols of the third (**G_3_OH**) and fourth (**G_4_OH**) generations were chosen (Figure 1) as reducing agents and stabilizers for the polyol process. The choice of polyols is due to their high biocompatibility (LD_50_~2000 mg/kg) [33] and a significant number of peripheral hydroxyl groups. Additionally, it has been previously demonstrated that the presence of hydroxyl groups in the polyol enables its use as a bifunctional reagent for the polyol process [31].

### 3.1. Study of the Interaction of Co^2+^ Ions with Polyol

The initial stage of metal ion association with the polyol matrix is critical for polyol synthesis. It determines the pre-organization and spatial localization of metal nanophases during nucleation, as well as the morphology and properties of the resulting nanocomposites. Spectrophotometry and NTA data were used to investigate the formation of the [Co^2+^–**G_n_OH**] (n = 3, 4) conjugate system. We conducted experiments to determine the optimal Co^2+^/**G_n_OH** ratio and to achieve maximum metal ion loading in the metal–polymer nanocomposite. The choice of DMSO as the dispersion medium at this stage of the research is dictated by its significance in biomedicine and by the methods used for the synthesis and subsequent application of the nanocomposites [34].

**Figure 2:**
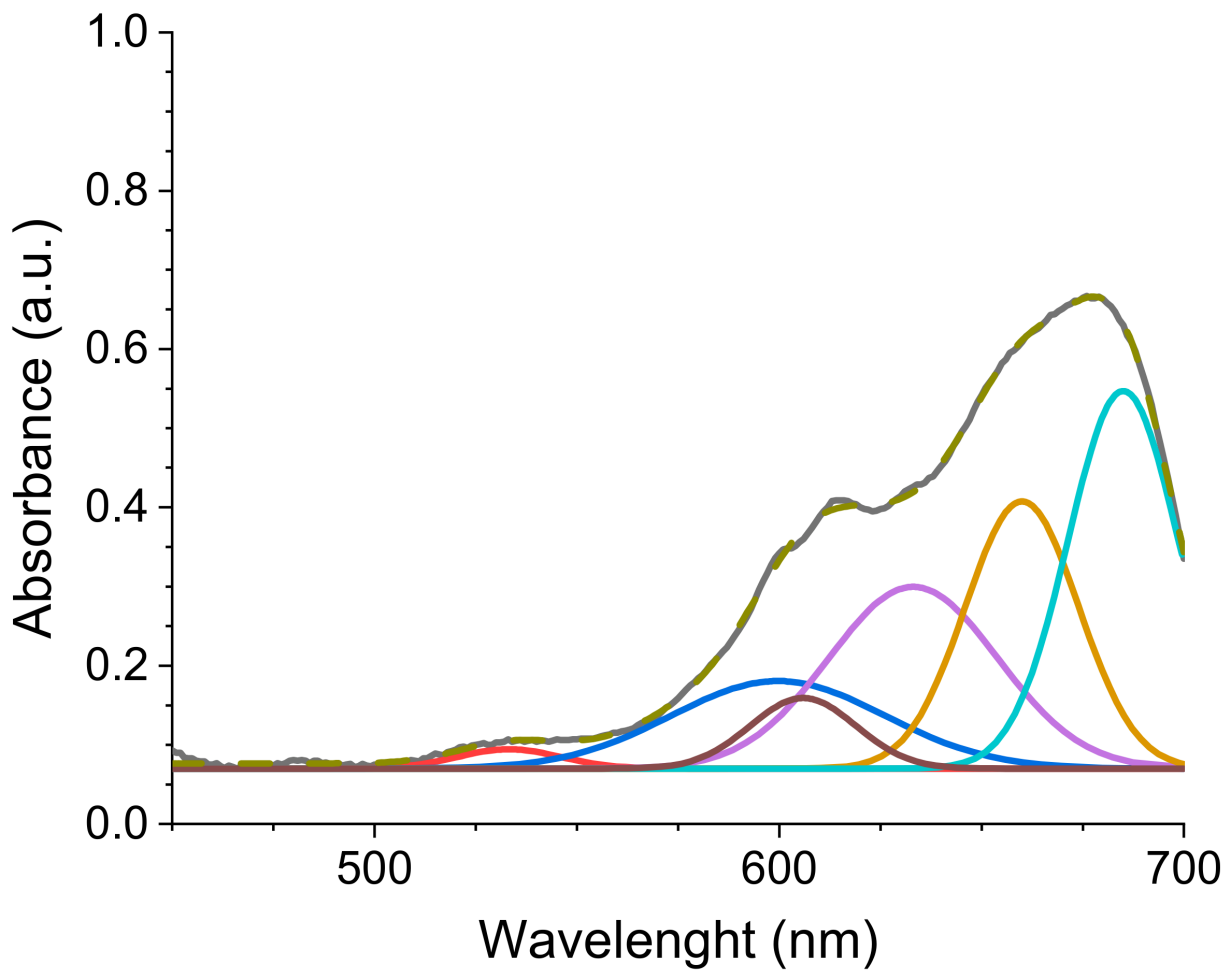
Band-resolved UV-Vis spectra of the mixture CoCl_2_-G_3_OH (C_CoCl2_ = 0.05 mol·L^−1^, C**_G3OH_** = 0.125 mg·mL^−1^).

The UV-Vis spectrophotometry data for the [CoCl_2_–**G_3_OH**] and [CoCl_2_–**G_4_OH**] systems are identical and are presented in Figure 2 and Figure S1. In the initial system upon the addition of solid CoCl_2_·6H_2_O to a solution of the polyol **G_n_OH** in DMSO, cobalt–DMSO complexes of the type [Co(DMSO)_6_]^2+^ are present. During the pre-organization stage, the solvent molecules in the cobalt coordination sphere are replaced by the hydroxyl groups of the polyol. The decrease in the intensity of the [Co(DMSO)_6_]^2+^ band at 533 nm and the appearance of several additional absorption bands at 633 and 659 nm correspond to the formation of coordinatively saturated mixed-ligand CoCl_x_(OH)_y_ centers, in which the cobalt ion is bonded to the polymer via a terminal hydroxyl group [35]. The expected hypsochromic shift in the bands is observed as the number of hydroxyl groups in the first coordination sphere increases. This confirms the formation of coordinatively saturated metal centers [CoCl_2_(OH_polyol_)_2_] [35] as early as the reagent-mixing stage. To evaluate the thermodynamics of this process and the stoichiometry of the resulting coordination centers in detail, it is necessary to perform quantum-chemical calculations for the Co^2+^–DMSO and Co^2+^–**G_n_OH** systems. These results will be presented in our future publication.

A quantitative assessment of the parameters for Co^2+^ ion immobilization by the polyol **G_n_OH** (n = 3, 4) was performed based on static sorption data (Figure S2). Analysis of the experimental data using the Langmuir model enabled the calculation of the binding constant (K_L_) and the maximum sorption capacity (A_∞_) (Table 1) at the pre-organization stage. It is noteworthy that, despite a two-fold increase in the number of peripheral hydroxyl groups when transitioning from **G_3_OH** (32 OH) to **G_4_OH** (64 OH), the maximum sorption capacity increases by only ~15%, whereas the specific capacity per OH group for **G_4_OH** (~0.050) drops by nearly half compared to **G_3_OH** (~0.087).

The dispersion characteristics (mean hydrodynamic diameter d_h_^mean^, hydrodynamic diameter of the main fraction d_h_^mode^, and particle concentration C_NP_) of solutions containing the initial polyols and their complexes with Co^2+^ were evaluated using the NTA method (Table 1, Figure S2). The degree of polydispersity (PDD) was calculated as the ratio d_h_^mode^/d_h_^mean^. A PDD value of ~1 indicates particle monodispersity. It has been established that G_3_OH and G_4_OH molecules form monodisperse aggregates [G_n_OH]_x_ with average hydrodynamic diameters of 106 and 134 nm, respectively. These aggregates are formed through intra- and intermolecular hydrogen bonds between terminal hydroxyl groups. The two generations of polyols exhibit fundamentally different behavior upon saturation of the aggregates with cobalt ions. In the [Co^2+^–**G_3_OH**] system, the hydrodynamic diameter increases to 282 nm, the particle concentration drops 23-fold, the distribution becomes polydisperse, and the PDD decreases to 0.58. This indicates a surface-type localization of the Co^2+^ ion driven by coordination interactions with the terminal OH groups of **G_3_OH**. The NTA parameters point to intermolecular coordination of metal ions. In this case, Co^2+^ ions act as interparticle “bridges”, leading to the formation of metal–polymer associates. In the [Co^2+^–**G_4_OH**] system, the hydrodynamic diameter remains virtually unchanged relative to the initial polymer aggregates, and the system retains the PDD value and particle concentration characteristic of the **G_4_OH** polymer (Table 1). This indicates an intracavity localization of the Co^2+^ ion within the [Co^2+^–G_4_OH] system.

Differences in cobalt ion binding by polyols of various generations may be attributed to polymer architectural parameters. Dendritic-like polyester polyols possess a hydrophobic polyester core and a hydrophilic shell composed of terminal and linear hydroxyl groups. The latter forms a region where polar groups are localized. The key characteristic determining the contrasting trends in metal ion localization across polyols of different generations is a structural parameter—the degree of branching (DB) of the dendrimers and dendritic-like polymers. According to calculations based on ^1^H NMR spectroscopy data, the degree of branching (DB) increases with the generation, reaching 0.37 for G_3_OH and 0.48 for **G_4_OH** (Figure S3, Table 1). This implies that the **G_3_OH** polymer has a more “open” architecture with greater mobility and accessibility of the terminal OH groups for the surface coordination of metal ions. For **G_4_OH**, the higher DB indicates a compact architecture with a distinct hydrophobic polyester core. An increase in the density of peripheral OH groups creates steric hindrance for the surface coordination of cobalt ions. Consequently, hydrophilic Co^2+^ ions preferentially coordinate with linear OH groups located within pre-organized “polar pockets” at the core–shell interface of the polyol adopting an intracavity localization mode. Such coordination does not induce interparticle aggregation. Instead, it compacts the macromolecular coil while preserving the monodisperse nature of the distribution. The transition from surface localization to localization within “polar pockets” results in only a slight increase in the sorption capacity for [Co^2+^–**G_4_OH**], as the number of such binding sites is limited. A slight increase in the binding constant K_L_ may be attributed to the formation of a more favorable microenvironment and reduced competition from DMSO and chloride ions.

### 3.2. Study of Hyperbranched Polyol Process for Synthesis of Co/G_n_OH Nanocomposites

The hyperbranched polyester polyols must act as a bifunctional agent (a reducing agent for cobalt ions and a stabilizer for cobalt clusters and nanoparticles) during the polyol process in a two-component medium. To establish the concentration conditions of the hyperbranched polyol process (HB-polyol process) in a two-component system, the cobalt-containing nanocomposites were synthesized at various ν_Co2+_/ν_OHpolyol_ molar ratios. The synthesis of cobalt-containing nanocomposites was carried out in a **G_n_OH** solution at a constant **G_n_OH** concentration and a varying CoCl_2_ concentration. The HB-polyol process was carried out by heating to 240 °C at 8 h. The formation of the nanocomposite **Co/G_n_OH** was accompanied by the following color transitions: blue–blue–green (Figure S4). All synthesized samples have high colloidal stability.

**Figure 3:**
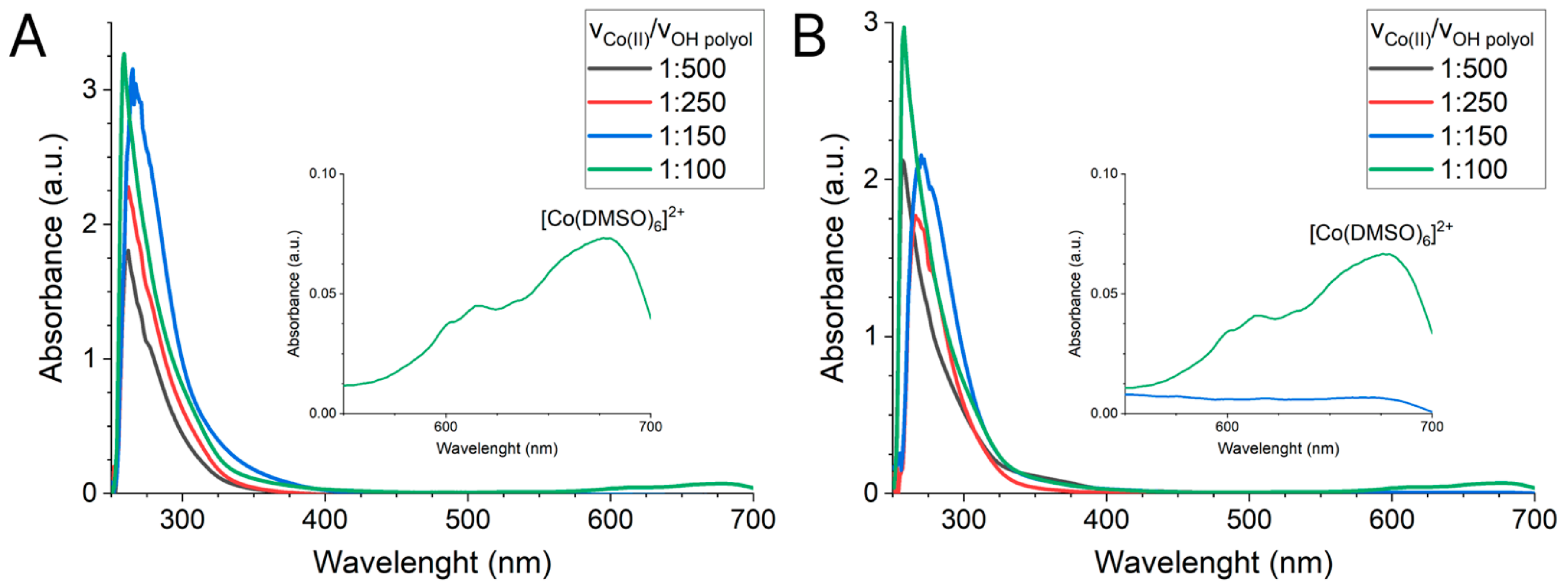
UV-Vis spectra of dispersions **Co/G_3_OH** (**A**) and **Co/G_4_OH** (**B**) (C_Co/GnOH_ = 2 mg/mL) in DMSO.

Eight hours after the start of synthesis, the UV-Vis spectra of all **Co/G_n_OH** samples contain an intense plasmon resonance band of Co^0^ clusters at λ_SPR_ = 273–279 nm (Figure 3) [36]. Also, in the spectra of the samples, there is a weak shoulder in the region of 343–367 nm, in accordance with the plasmon resonance band of nanosized cobalt oxide Co_x_O_y_ on the surface of the metal particles [37]. This may be due to partial oxidation of the surface of Co(0) nanoclusters by dissolved O_2_. A group of absorption bands in the region of 600–700 nm related to d-d transitions of unreduced polymer-coordinated Co^2+^ ion is observed in the spectrum of samples synthesized at the molar ratio of ν_Co2+_/ν_OHpolyol_ = 1:100. Thus, the bifunctional (reducing and stabilizing) activity of hyperbranched polyesters of the third and fourth generations in the polyol process for producing cobalt nanoparticles is limited by the ratio ν_Co2+_/ν_OHpolyol_ = 1:150. Therefore, **Co/G_n_OH-4** composites synthesized at the molar ratio ν_Co2+_/ν_OHpolyol_ = 1:100 were not studied in further work.

The evolution and stabilization of the characteristics of **Co/G_n_OH** nanosystems after cooling were evaluated using combined UV-Vis analysis (Figure S5) and NTA (Figure S6).

**Figure 4:**
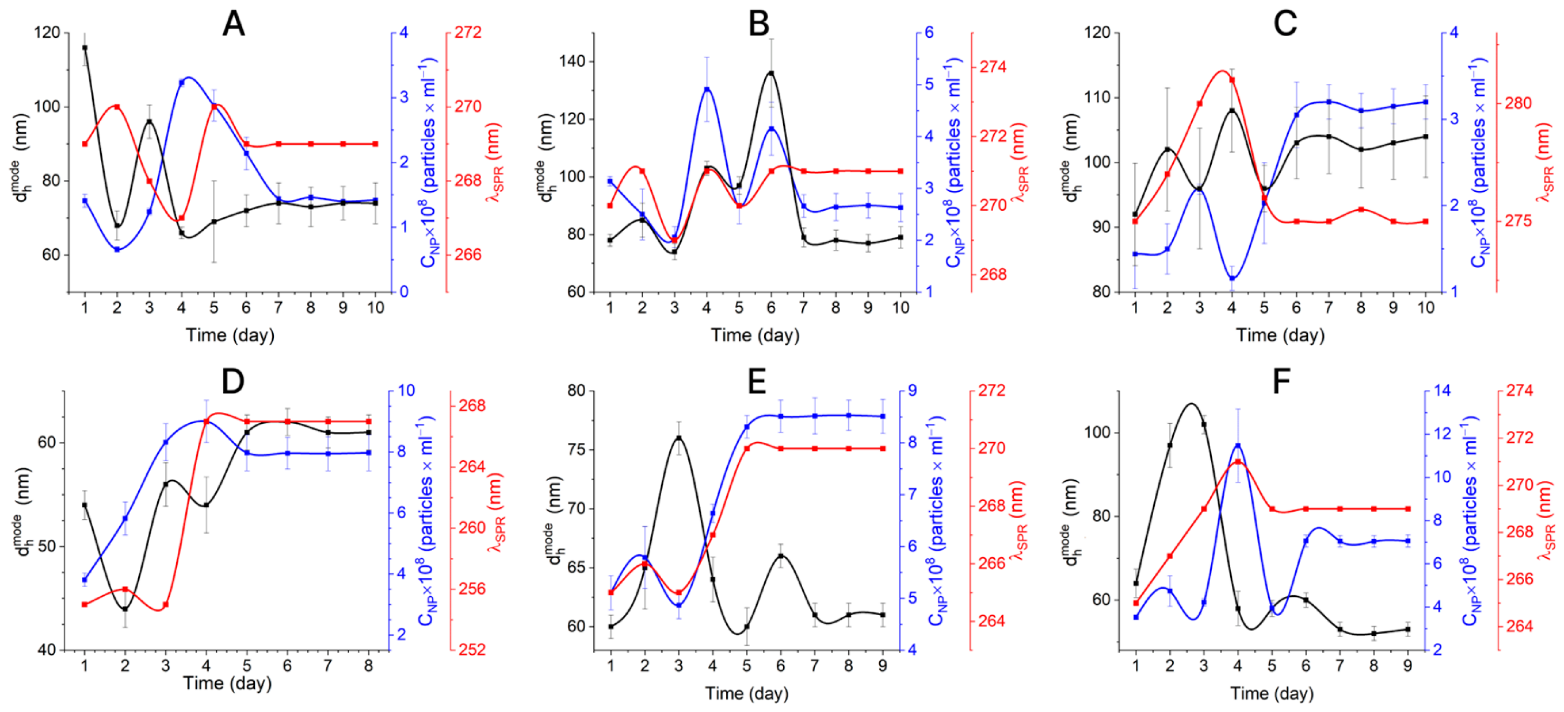
Profiles of the mode hydrodynamic diameter (d_h_^mode^, black line), concentration of particles (blue line) and λ_SPR_ (red line) for **Co/G_3_OH-1** (**A**), **Co/G_3_OH-2** (**B**), **Co/G_3_OH-3** (**C**), **Co/G_4_OH-1** (**D**), **Co/G_4_OH-2** (**E**), and **Co/G_4_OH-3** (**F**) samples in situ.

It has been established that the shift in the surface plasmon resonance band maximum of Co^0^ nanoclusters (λ_SPR_ = 273–279 nm) directly correlates with pronounced oscillations in the mean hydrodynamic diameter (d_h_^mode^) during the early stages of stabilization (Figure 4). For 1–3 days after cooling, the nanosystems remain unstable due to competing processes, namely residual nucleation, cobalt nanoparticle growth and subsequent coordination stabilization by the polyol matrix. The plateauing of the λ_SPR_ value indicates that the formation of a colloidally stable nanophase is complete by the fifth day (for **Co/G_4_OH**) and the seventh day (for **Co/G_3_OH**) following the completion of synthesis and the cooling of the reaction mixture. This means that the key redox and structure-forming processes are complete, the rate of maturation of the metal nanophase becomes negligibly low, and thermodynamic equilibrium is reached between the metal nanophase and the polymer matrix.

A joint analysis of spectrophotometry and NTA data (Figure 4) made it possible to draw conclusions regarding the stabilization (“maturation”) mechanism of **Co/G_n_OH** composite nanoparticles. In most cases, **Co/G_3_OH** nanocomposites retain the polydispersity characteristic of the initial [Co^2+^-G_3_OH] ion–polymer system. Stabilization in this system proceeds primarily via the mechanism of direct Ostwald ripening [9,38], which involves particle enlargement resulting from the dissolution of smaller clusters and the transport of metal atoms to larger crystallization centers on the macromolecule surface. Increasing the concentration of the CoCl_2_ precursor enhances the surface doping of the polymer with cobalt clusters, which manifests as an increase in hydrodynamic diameter across the series: 74 nm (**Co/G_3_OH-1**), 79 nm (**Co/G_3_OH-2**), and 103 nm (**Co/G_3_OH-3**). Subsequent aggregation driven by hydrogen bonding between the peripheral groups of **G_3_OH** impairs the dispersity of the nanosystem.

The reduction in cobalt ions pre-organized within the “polar pockets” of **G_4_OH** leads to the formation of monodisperse metallopolymer nanocomposites containing dendrimer-encapsulated metal nanoparticles (DENs). In the **Co/G_4_OH** system, a change in the ripening mechanism is observed as the precursor salt concentration increases. At low metal loading in **Co/G_4_OH-1** (molar ratio ν_Co_^2+^:ν_OH_ = 1:500), the process also follows the Ostwald ripening mechanism but is confined by the cavity volume, resulting in a D_h_ value of ~147 nm. Increasing the metal loading to molar ratios of 1:250 (**Co/G_4_OH-2**) and 1:150 (**Co/G_4_OH-3**) triggers a transition to the digestive ripening mechanism [39,40,41]. Under conditions of nanoconfinement in the hydrophobic core of the dendrimer-like macromolecule, cobalt atom redistribution occurs. It leads to the dispersion of large nuclei and the formation of smaller close-packed nanoclusters. This is evidenced by a decrease in the hydrodynamic diameter from 147 nm (**Co/G_4_OH-1**) to 66 nm (**Co/G_4_OH-2**) and 53 nm (**Co/G_4_OH-3**). This effect is a characteristic feature of DEN synthesis within the nanoconfined cavities of dendritic structures [42,43].

### 3.3. IR-Study of G_n_OH Oxidation

**Figure 5:**
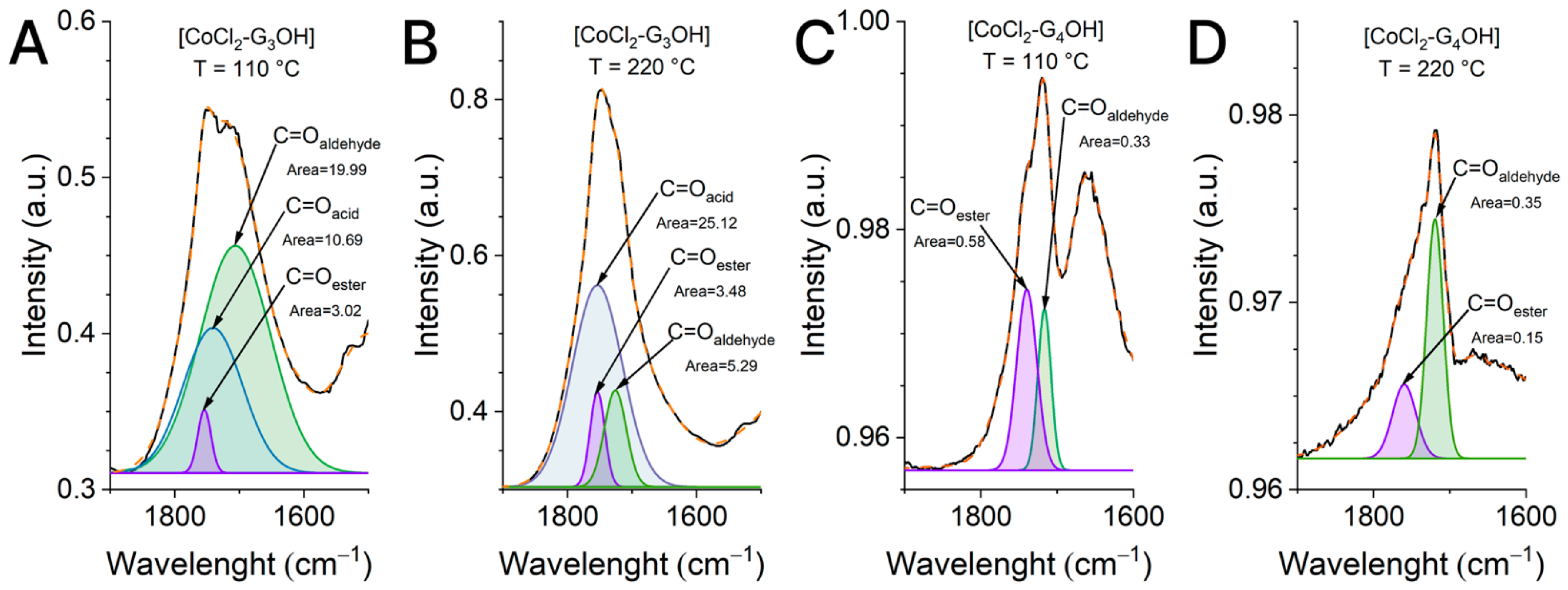
FT-IR spectra of the [CoCl_2_-**G_n_OH**] mixture at 110 °C (**A**,**C**) (R^2^ = 99.9, χ^2^ = 6.14·10^−6^), 220 °C (**B**,**D**) (R^2^ = 99.9, χ^2^ = 4.96·10^−6^), and interpolation of the absorption band from the Gauss distribution function.

The oxidation process of the hyperbranched polyester polyol **G_n_OH** and the formation of a stabilizing shell on cobalt nanoparticles were investigated in situ using diffuse reflectance infrared spectroscopy in the temperature range of 35–220 °C (Figure 5, Figures S7 and S8). Initial studies regarding the fourth-generation polyol **G_4_OH** were presented in the article [31]. This study established that the oxidation of the terminal hydroxyl groups of the hyperbranched polyester polyols **G_n_OH** begins as early as the heating stage of the reaction mixture. A concomitant systematic decrease is observed in the intensity of the stretching vibrations ν(OH_alcohol_) at ~3439 cm^−1^ and the bending vibrations δ(OH_alcohol_) at ~1314 cm^−1^ (Figure S8). The general oxidation process of OH groups for **G_4_OH** begins at a lower temperature (90 °C for **G_4_OH** versus 100 °C for **G_3_OH**). This may be attributed to the higher content of peripheral functional groups in the fourth-generation polyol (64 groups) and the high local concentration of active sites available for coordination with Co^2+^ ions and for initiating the redox process via an acid-base catalysis mechanism.

To identify oxidation pathways, the ν(C=O) band profile in the 1900–1500 cm^−1^ range was mathematically processed using a Gaussian distribution function approximation, including the use of the areas of the corresponding signals after deconvolution for quantitative assessment (Figure 5). Fundamental differences were established regarding the oxidation of the polyol’s OH groups and the chemical composition of the polymer surface in the resulting nanocomposites. Upon heating the [CoCl_2_–**G_3_OH**] system to 110 °C, components corresponding to the stretching vibrations of aldehyde carbonyl groups (ν(C=O_aldehyde_) ~1720 cm^−1^, signal area 19.99) and carboxylic acid carbonyl groups (ν(C=O_acid_)~1748 cm^−1^, signal area 10.69) appear in the spectrum. Raising the temperature to 220 °C leads to a dramatic transformation: the area of the ν(C=O_aldehyde_) band decreases to 5.29, whereas the contribution of ν(C=O_acid_) increases to 25.12. This indicates the two-stage oxidation of the terminal alcohol groups of the **G_3_OH** polyol following pathway of alcohol → aldehyde → carboxyl groups [9].

However, in the [CoCl_2_–**G_4_OH**] system, the spectral pattern remains unchanged throughout the entire heating range (110–220 °C) and is consistent with the formation of an aldehyde group as the dominant oxidation product. The areas of the C=O_aldehyde_ signals are 0.33 and 0.35 at 110 °C and 220 °C, respectively. There is no contribution from carboxyl groups. Thus, the oxidation of the alcohol groups of the **G_4_OH** polyol stops at the alcohol-to-aldehyde stage.

To confirm that the observed oxidation of the terminal hydroxyl groups of the polyol is indeed driven by the polyol process in the presence of cobalt species, a control experiment was performed. The hyperbranched polyol **G_n_OH** was subjected to heating following the same temperature program in DMSO medium, but in the absence of CoCl_2_ salt and NaOH. According to in situ FT-IR spectroscopy (Figure S9), no changes in the carbonyl stretching region (1900–1500 cm^−1^) were observed for the pure polyol over the entire investigated temperature range. Thus, the formation of aldehyde and carboxyl groups during the synthesis is directly associated with the oxidation of hydroxyl groups that provide electrons for the reduction of Co^2+^ ions, and is not a consequence of thermal treatment alone.

Additionally, the appearance and increasing intensity of bands at 668 cm^−1^ and 579 cm^−1^—assigned to ν(Co(II)-O) and ν(Co(III)-O) stretching vibrations [44] in cobalt oxide— were observed in the spectra of both systems. This confirms the formation of a Co_x_O_y_ oxide shell on the surface of the reducing Co^0^ clusters under aerobic conditions.

The observed difference in oxidation pathways correlates with the localization of the metallic nanophase as determined by UV-Vis and NTA data. In the **Co/G_3_OH** system, the metal nanophase is predominantly localized at the HB-polyol periphery. Cobalt clusters forming on the surface can act as an effective heterogeneous catalyst for the oxidation of the OH groups of the hyperbranched polyol **G_3_OH**. This hypothesis is supported by literature data regarding the ability of cobalt and cobalt oxide nanoparticles to catalyze the dehydrogenation of alcohols to aldehydes [45,46] and also accelerate the further oxidation of aldehydes to acids through the transfer of reactive oxygen species [47,48]. In this case, a high concentration of Co^0^/Co_x_O_y_ particles at the periphery of **G_3_OH** ensures a more complete conversion of the polymer’s terminal OH groups during the redox process.

In the **Co/G_4_OH** system, the cobalt clusters being formed are localized within the hydrophobic core of the polymer. A steric barrier shields the clusters from direct contact with the external environment and the terminal OH groups on the surface, thereby blocking the catalytic action of the cobalt nanoparticles on the subsequent oxidation of the aldehyde. Consequently, the oxidation of **G_4_OH** concludes with the formation of aldehyde groups only.

Subsequently, the oxidized form of hyperbranched polyol **G_n_OH** acts as an effective macromolecular stabilizing ligand for cobalt nanoclusters. The resulting FT-IR spectra (Figure S10) indicate the involvement of carboxyl and aldehyde groups of **G_3_OH** and **G_4_OH**, respectively, in the stabilization of Co/Co_x_O_y_ nanoclusters through donor–acceptor interactions with coordinatively unsaturated surface cobalt atoms or Co^2+^/Co^3+^ ions at lattice defects.

### 3.4. Morphology of Co/G_n_OH Nanocomposite

**Figure 6:**
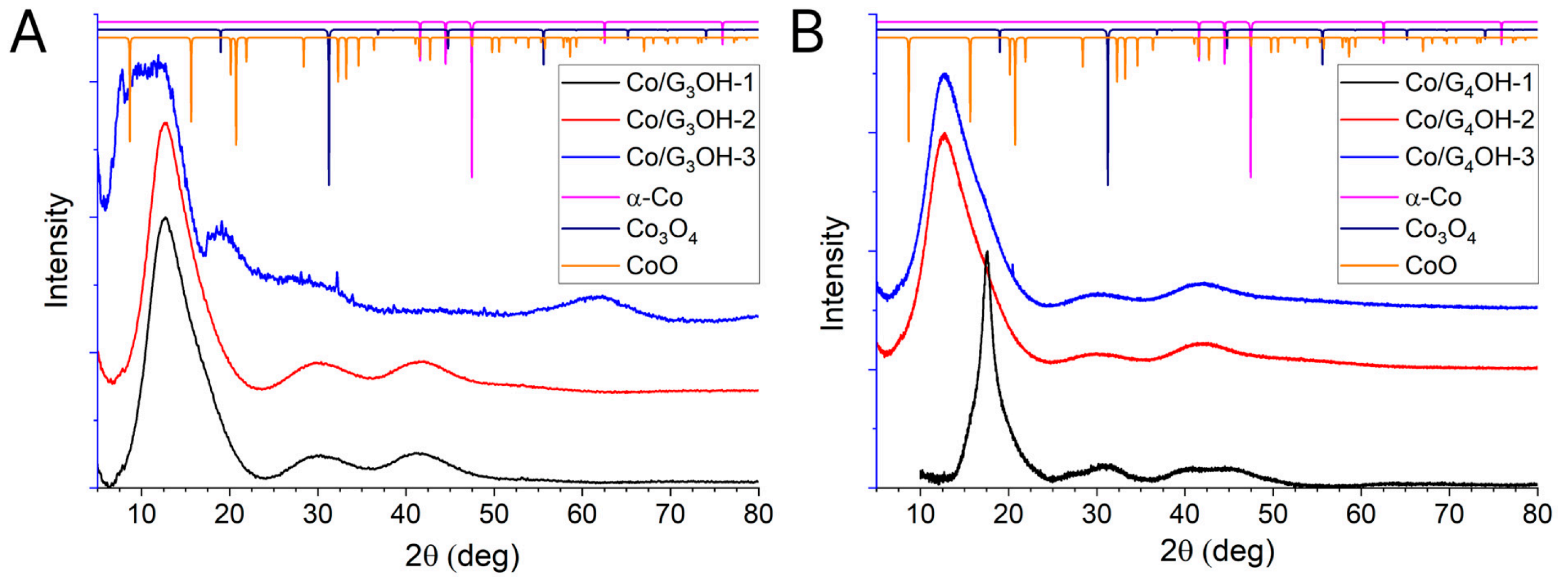
Diffractograms of samples of **Co/G_3_OH** (**A**) and **Co/G_4_OH** (**B**).

The phase composition of the produced **Co/G_n_OH** nanocomposites was assessed by using X-ray phase analysis. Qualitative analysis shows that all samples of cobalt **Co/G_n_OH** nanocomposites contained a reflection of the polymer matrix at 2θ equal to 17.69°. The signals at angles 2θ equal to 41.48° and 62.55° correspond to α-Co^0^ metal nanoparticles in all **Co/G_n_OH** samples (Figure 6). Based on the UV-Vis, FT-IR and XRD data, it can be assumed that the metal phase of the composite is represented by Co^0^ nanoparticles with Co_x_O_y_ oxides on their surface.

**Figure 7:**
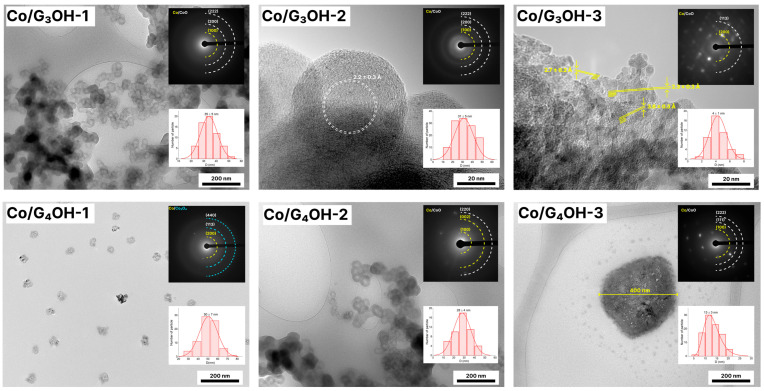
TEM images, particle size distributions, and electron diffraction of Co/G_3_OH and **Co/G_4_OH**.

The morphology of synthesized **Co/G_n_OH** was studied by transmission electron microscopy (TEM) with electron diffraction (Figure 7 and Figure S11, Table 2). TEM analysis revealed the formation of spheroidal polymer–metal composite nanoparticles in all **Co/G_n_OH** samples. Calculated interplanar spacing values indicate the presence of a metallic Co^0^ phase (Fm3m face-centered cubic crystal lattice), with CoO and Co_3_O_4_ oxides on the surface in all the composites (Figure 7).

For **Co/G_3_OH** dendrimer-stabilized nanoparticles (DSNs), an increase in cobalt loading results in a decrease in the average particle diameter, alongside an increase in the nanomaterial’s crystallinity. In the case of **Co/G_3_OH-1,** where the polymer is present in excess, “nanocomposite vesicles” are formed. In the inner volume, there are polymer molecules associated due to intermolecular hydrogen bonds of terminal partially oxidized hydroxyl and carboxyl groups, –C=O_acid_…H-O-_acid_, –C=O_acid_…H-O-_alcohol_, H-O-_alcohol_-H-O-_alcohol_, while composite **Co/G_3_OH** particles are localized within the shell. The formation of layer-by-layer radially oriented **Co/G_3_OH-2** nanostructures is observed as the metal loading increases. It can be hypothesized that the interlayer association in metal–polymer composites is carried out by surface concentrically arranged carboxyl and hydroxyl groups of the polymer, which are not alloyed cobalt nanoclusters. At the molar ratio ν_Co2+_/ν_OHpolyol_ = 1:150 in the **Co/G_3_OH-3** nanocomposite, metal clusters fully doped the surface groups of the polymer and blocked its association. Therefore, the particle size of the composite decreases to 4 nm. A transition to a core–shell structure is likely occurring, wherein polymer macromolecules passivate the cobalt nanoparticle surface, and supramolecular interactions between surface groups give rise to the “flaky” layered structures observed via TEM.

The formation of dendrimer-encapsulated nanoparticles (DENs) in **G_4_OH** proceeds via a fundamentally different mechanism. Due to the localization of the metal phase within the hydrophobic core of the macromolecule, the cobalt clusters do not cross-link the polymer chains. Association processes are governed primarily by the activity of unoxidized alcohol groups as this capability is significantly lower for aldehyde groups. TEM images (Figure 7) reveal isolated compact nanovesicles. The totality of the experimental data allows us to make the assumption that with an increase in the metal load in the series **Co/G_4_OH-1, -2, -3**, the macromolecular coil becomes denser due to the “compactification effect” [42,43] which is accompanied by an increase in the crystallinity of the metallic nanophase and a reduction in particle size (as determined by TEM) from 50 nm to 28 nm and 13 nm, respectively.

Data from the NTA of **Co/G_n_OH** metal–polymer nanocomposite nanoparticles (Figure S12, Table 2) fully confirm the patterns observed via TEM. It was found that increasing the metal loading leads to an increase in particle concentration in all cases. However, the change in the mean hydrodynamic diameter (d^h^_mean_) of the **Co/G_3_OH** and **Co/G_4_OH** particles follows an opposite trend. The formation of compact supramolecular architectures results in a reduction in the hydrodynamic diameter of the **Co/G_3_OH-1** and **Co/G_3_OH-2** composite particles to 74–79 nm compared to the pristine **G_3_OH** polymer.

Further intensification of surface doping of the polymer with increasing cobalt loading returns the hydrodynamic diameter of **Co/G_3_OH-3** particles to a value close to that of the pristine polymer (103 ± 6 nm). Notably, the **Co/G_3_OH-2** sample (featuring an ordered layered nanostructure) is the only monodisperse one in this series.

The **Co/G_4_OH** series is characterized by a monotonic decrease in particle hydrodynamic diameter as metal loading increases: from 149 nm for Co/G_4_OH-1 to 66 nm for **Co/G_4_OH-2** and 53 nm for **Co/G_4_OH-3**. The encapsulation of cobalt nanoparticles leads to compaction of the macromolecular coil (the “nanoconfinement” effect), which correlates with a reduction in the number of unoxidized OH groups participating in the hydrogen-bonding network across the **Co/G_4_OH-1, -2, and -3** series. Unlike **Co/G_3_OH**, all **Co/G_4_OH** samples retain a monodisperse distribution. This confirms the absence of intermolecular cross-linking and the stabilization of particles exclusively within the hydrophobic cavity of the dendrimer-like polymer.

It is important to note that the dispersity of the synthesized metal–polymer nanosystems (according to NTA data) fully corresponds to that of the respective initial polyols and ion–polymer nanohybrids at the pre-organization stage (Table 1). This unequivocally indicates the inheritance of the polymer matrix’s structural parameters during the formation of the final nanocomposite. An exception is the **Co/G_3_OH-2** sample, which, as shown above, forms radially oriented layered nanostructures; this explains the system’s transition to a monodisperse state.

### 3.5. Functional Properties of Co/G_n_OH Nanocomposites

#### 3.5.1. Thermal Stability

**Figure 8:**
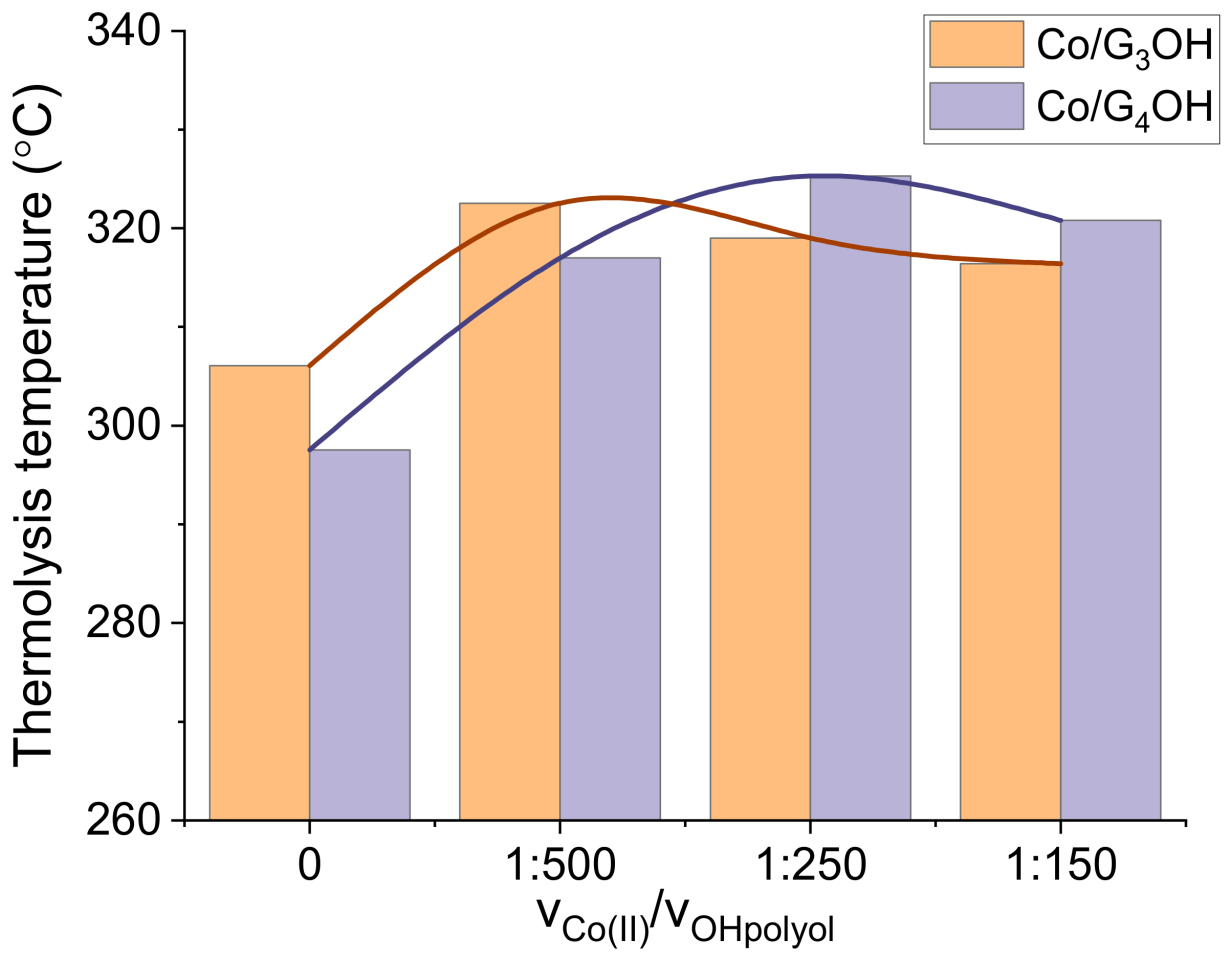
Dependence of thermolysis temperature on the loading of the metallic nanophase for **Co/G_n_OH** nanocomposites.

The thermal stability of **G_n_OH** polyols and nanocomposites based on them was evaluated by thermogravimetry. According to TG-DTG analysis, **G_n_OH** polyols are stable in the range of 25–300 °C (Figure 8 and Figure S13). The primary mass loss associated with the dehydration of hydroxyl groups and the degradation of the polyester core is observed at temperatures above 350 °C, which is in good agreement with literature data for bis-MPA-based hyperbranched polyols [49]. The presence of cobalt in the metal–polymer nanocomposites leads to an increase in the thermolysis temperature by 10–16 °C for **Co/G_3_OH** and 30–36 °C for **Co/G_4_OH**.

#### 3.5.2. Magnetic Properties

Cobalt-based nanocomposites are attractive for magnetic-based biomedical applications because metallic Co exhibits a high Curie temperature (*T_c_* ≈ 1390 K for the bulk material) and a large saturation magnetization, *M*_s_ ≈ 1440 emu cm^−3^ or *B_s_* = *μ*_0_*M*_s_ ≈ 1.8 T, when expressed as a magnetic flux density. In the nanoscale regime, the Curie temperature is usually reduced (typically to 1000–1300 K), owing to surface disorder, and the measured *M_s_* can be 10–30% lower than the bulk value because of partial oxidation.

The spinel oxide Co_3_O_4_, which is formed on the surface of the particles during synthesis, is an antiferromagnet with a Néel temperature *T_N_* ≈ 40 K (the related oxide CoO orders at ≈291 K). Consequently, the magnetic response of the nanocomposites is a combination of a ferromagnetic (or superparamagnetic) metallic Co core and a weakly magnetic Co_3_O_4_ shell.

**Figure 9:**
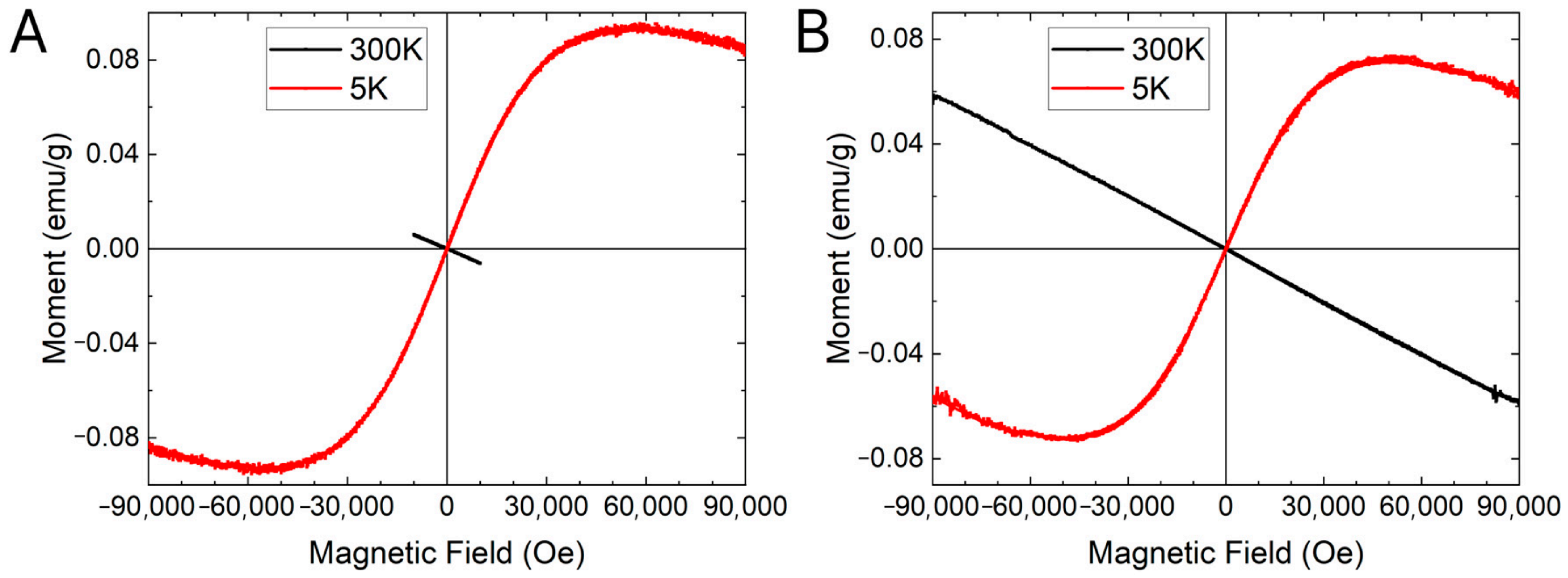
Magnetization versus applied field for (**A**) **Co/G_3_OH-2** and (**B**) **Co/G_3_OH-3**, measured at 300 K (black symbols) and 5 K (red symbols).

The field-dependent magnetization of the **Co/G_3_OH** nanocomposites was measured at several temperatures. Reliable data were obtained for the two samples that contain the highest cobalt loadings, **Co/G_3_OH-2** and **Co/G_3_OH-3**, and are shown in Figure 9.

At 5 K, both samples display a steep, nearly saturating increase in magnetization with field, characteristic of blocked superparamagnetic (ferromagnetic) particles. The response is not linear; therefore, the materials cannot be described as paramagnetic at this temperature. Upon heating to 300 K, the magnetic moments of the Co cores become thermally randomized; the superparamagnetic contribution diminishes and the temperature-independent diamagnetic signal of the polymer matrix becomes more apparent. The diamagnetic contribution was evaluated from the high-field linear part of the 300 K curves and expressed as a mass susceptibility χ_0_ (Table 3). The magnetization was then corrected according to

$$M_{\mathrm{corr}}\left(H\right)=M_{\mathrm{raw}}\left(H\right)-\chi_{0}H,$$

where H is the applied field (in Oe) and M is expressed in emu g^−1^.

**Figure 10:**
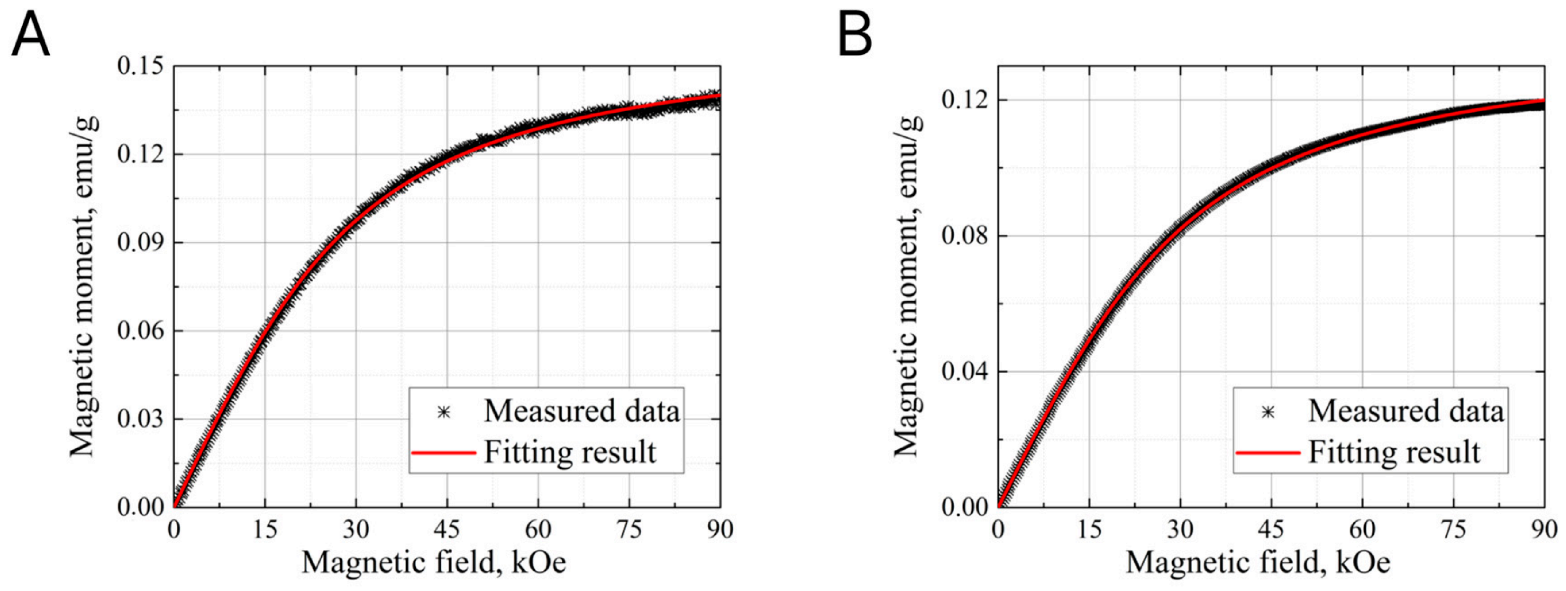
The corrected low-temperature magnetization curves versus applied field for (**A**) **Co/G_3_OH-2** and (**B**) **Co/G_3_OH-3** at 5 K (open black circles). Red lines are Langevin-function fits using the particle diameter as a fitting parameter.

The corrected low-temperature magnetization curves were fitted using the Langevin function [50]:

$$M_{L}\left(H\right)=M_{s}L\left(\frac{\mu H}{k_{B}T}\right),$$

$$L\left(x\right)=\coth x-\frac{1}{x},$$

which is appropriate for non-interacting, single-domain particles whose magnetic moment μ is fixed. In the fit, the magnetic-core diameter (d) and the average magnetic moment per particle (μ) were treated as independent parameters; the saturation magnetization of bulk Co (M_s_ ≈ 1440 emu cm^−3^ at RT or ≈ 162 emu g^−1^) was used to relate μ and d (μ = M_s_ · V, V = πd^3^/6). The fits are shown in Figure 10 and the resulting parameters are listed in Table 4.

The much smaller net moment observed for **Co/G_3_OH-3** reflects a higher fraction of the antiferromagnetic Co_3_O_4_ shell, which partially compensates for the metallic core magnetization.

The presence of a sizable superparamagnetic component at a physiological temperature (≈310 K) is a prerequisite for magnetic-guided drug delivery and magnetic hyperthermia.

#### 3.5.3. Biocompatibility

Hemocompatibility refers to the way in which a foreign substance interacts with blood, resulting in minimal negative effects. A material is considered hemocompatible when it results in less than 5% hemolysis [51,52,53].

**Figure 11:**
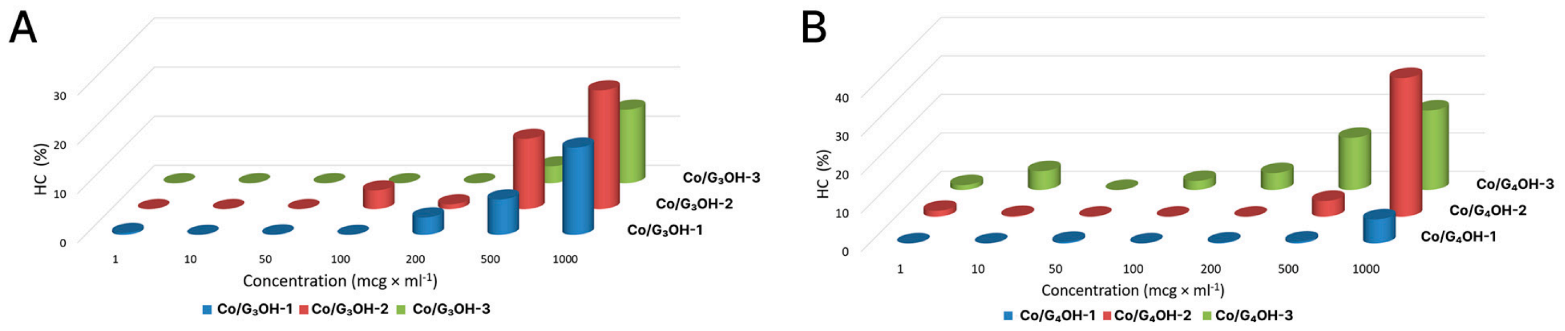
Effect of **Co/G_3_OH** (**A**) and **Co/G_4_OH** (**B**) and concentration on hemolysis (C_Co/GnOH_ = 1–1000 mcg × mL^−1^).

The biocompatibility of the **Co/G_n_OH** composite material was assessed in relation to the red blood cells of a healthy donor according to the method of [32], as the hemolysis coefficient (HC, %) and the HC_50_ value, which corresponded to the sample concentration at which 50% erythrocyte hemolysis was observed. Upon transfer of the **Co/G_n_OH** nanocomposites from DMSO to PBS, no critical changes in the colloidal state are observed. UV-Vis spectroscopy does not detect Co^2+^ ion release within 24 h, which confirms the stability of the coordination bonds between the polyol shell and the nanoparticle surface in neutral media. The data are shown in Figure 11 and Table 5. According to the literature, substances with a hemolytic activity of less than 20% are not potentially dangerous to the body and can be used for further developments in biomedicine and pharmacology. The parent polyols **G_3_OH** and **G_4_OH** demonstrated high hemocompatibility (HC_50_~17–18 mg/mL), whereas the precursor salt CoCl_2_ exhibited high toxicity (HC_50_ = 2.82 mg/mL). When assessing the hemolytic activity (HC, %) of **Co/G_n_OH** nanocomposites, it was found that **Co/G_n_OH** has low hemolytic activity in the concentration range of 1–1000 μg/mL, which allows its use for further biomedical developments. The value of the hemolysis coefficient corresponding to the destruction of 50% of erythrocytes—HC_50_—is presented in the table. For **Co/G_n_OH** samples, the HC_50_ value varies depending on the amount of metal phase present. An increase in metal phase loading leads to a decrease in the biocompatibility of the material.

An analysis of HC_50_ value demonstrates a relationship between hemocompatibility and the morphological and compositional characteristics of the nanocomposite polymer surfaces. The **Co/G_4_OH** system exhibits significantly higher HC_50_ values (8.89 and 7.83 mg/mL for samples **Co/G_4_OH-1** and **Co/G_4_OH-2**, respectively) compared to the **Co/G_3_OH** system (2.85 and 1.98 mg/mL for corresponding samples) at comparable metal loadings. This is attributed to the localization of the cobalt nanophase within the cavities of the **G_4_OH** polyol. The effective steric barrier provided by the polyester core and the layer of surface aldehyde groups shields erythrocytes from direct contact with toxic cobalt clusters and their oxidation products. In contrast, the peripheral localization of nanoparticles in **Co/G_3_OH,** accompanied by the formation of chemically active carboxylate groups, results in intense contact between the nanophase and the cell membrane. The result is a more pronounced hemolytic effect.

The anomalously low hemocompatibility (HC_50_~1.4 mg/mL) observed for the **Co/G_3_OH-3** and **Co/G_4_OH-3** samples with maximum metal loading is attributed to a combination of two factors identified via TEM and NTA data (Table 2). First, the **Co/G_3_OH-3** and **Co/G_4_OH-3** nanocomposites consist of small-sized particles with diameters of 4 ± 1 nm and 13 ± 3 nm, respectively. According to the literature, nanoparticles smaller than 10 nm can readily penetrate cell membranes and induce the generation of reactive oxygen species (ROS), leading to oxidative damage of the erythrocyte lipid bilayer and subsequent cell destruction [54,55]. In the case of **Co/G_3_OH-3**, this effect is intensified by the maximum surface loading of the metal at the polymer’s periphery. Second, for the **Co/G_4_OH-3** sample, the formation of large agglomerates (400 nm in size) was observed (TEM data, Figure 7) despite the intracavity nature of the stabilization. Such large polymer conglomerates filled with cobalt clusters can cause mechanical damage to the erythrocyte membrane upon contact (e.g., through shear stress or physical rupture), thereby initiating the non-oxidative pathway of hemolysis.

#### 3.5.4. Modulation of Enzymatic Activity

**Figure 12:**
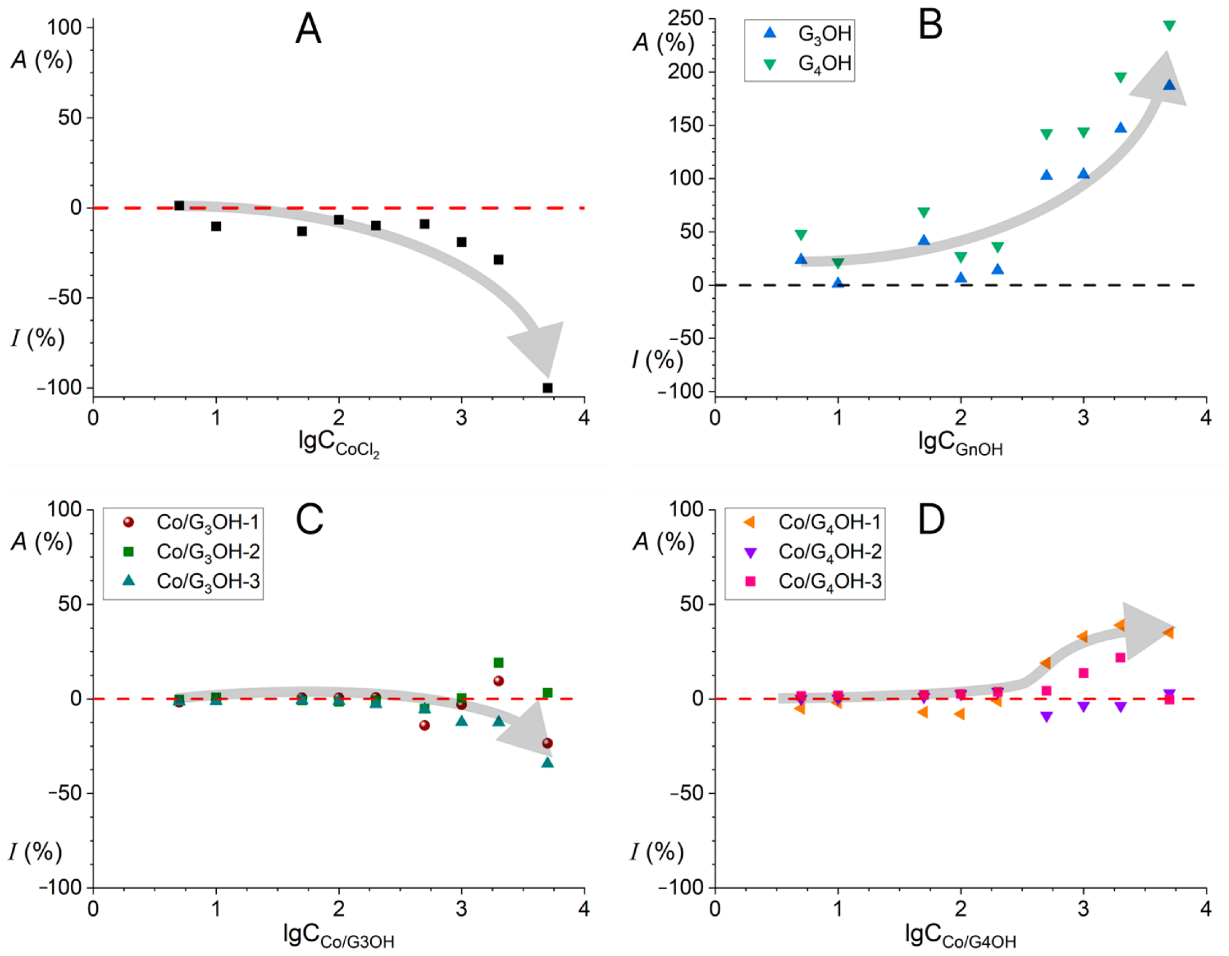
Enzymatic activity of chymosin in the presence of nanocomposites CoCl_2_ (**A**), **G_n_OH** (**B**), **Co/G_3_OH** (**C**), and **Co/G_4_OH** (**D**).

This study investigated the modulatory activity of CoCl_2_ salt, hyperbranched polyester polyols (**G_n_OH**), and synthesized **Co/G_n_OH** nanocomposites on the secretory aspartic proteinase chymosin, which was isolated from a culture of the mold fungus *Aspergillus niger* (Figure 12). It was found that CoCl_2_ exhibits an inhibitory effect that increases with concentration (Figure 12A), whereas GnOH polyols exert an activating effect (Figure 12B). Furthermore, the activating effect was observed to increase with the molecular weight and generation of the **G_n_OH** polyol.

It was found that **Co/G_n_OH** nanocomposite samples (Figure 12A) exhibit an inhibitory effect, while **Co/G_n_OH** nanocomposites (Figure 12B) exhibit an activating effect. Generally, it can be shown that increasing the molecular weight and polymer generation for **Co/G_n_OH** leads to a change in the direction of the antiproteinase activity effect. In the case of **Co/G_n_OH** nanocomposites (Figure 12C,D), it was found that the modulatory properties are determined by the localization of the cobalt nanophase, which, in turn, is governed by the architecture of the hyperbranched polyol. **Co/G_3_OH** nanocomposites, characterized by the surface localization of cobalt nanoparticles, exhibit an inhibitory effect (Figure 12C). This effect is most pronounced for the low-dimensional **Co/G_3_OH** particles, which show the lowest hemocompatibility. At the same time, **Co/G_4_OH** nanocomposites (Figure 12D), containing an encapsulated metal nanophase, exhibit a predominantly activating effect (Figure 12D). The **Co/G_4_OH-1** and **Co/G_4_OH-2** samples featuring particle diameters of 24–57 nm appear to be the most promising. Within the concentration range consistent with biomedical standards, they combine an activation effect with high hemocompatibility.

#### 3.5.5. Antimycotic Activity

To assess the potential of synthesized cobalt nanocomposites as antibacterial agents for suppressing proteinase-dependent, highly resistant pathogenic cultures (specifically *Candida albicans*, *Cryptococcus laurentii*, *Aspergillus fumigatus* and *Aspergillus niger*), the antimycotic effect of **G_n_OH** polyols and **Co/G_n_OH** nanocomposites was investigated using a disk diffusion test (Table 6, Figures S14 and S15). The study utilized polymer and nanocomposite samples at a concentration of 2 µg/mL, a level consistent with standards for biomedical applications. This concentration corresponds to a safe threshold level with a hemolysis coefficient (HC) of less than 5% for all synthesized **Co/G_n_OH** nanocomposites. Selecting a single concentration enables a comparative assessment of the biological activity of all synthesized samples under conditions guaranteed to be suitable for biomedical applications. It was determined that the polyols **G_3_OH** and **G_4_OH** themselves exhibit no antifungal activity against any of the tested cultures. This indicates that the biological activity is attributable solely to the metallic nanophase (Co^0^/Co_x_Oᵧ), which gives rise to cobalt ions and generates reactive oxygen species (ROS) that damage fungal cell walls.

In general, for **Co/G_n_OH** nanocomposites, increasing the metal nanophase content leads to an enlargement of the zone of lysis across all tests. However, the antifungal activity is determined not only by the total metal concentration but also by the nanocomposite’s morphological profile (particle size, degree of aggregation, and the localization pattern of the nanophase). Despite the fact that the open architecture of **G_3_OH** facilitates unobstructed contact between the cobalt nanoparticles and the fungal cell wall and that carboxylate groups promote adhesion to the surface, thereby enhancing local action, **Co/G_3_OH** samples exhibit no effect on *Aspergillus fumigatus* (zone of lysis is 0 mm). However, **Co/G_4_OH-2** and **Co/G_4_OH-3** exhibit activity (10.15 and 9.82 mm, respectively). This indicates that the dense cell wall of *Aspergillus fumigatus* acts as a formidable barrier to the large peripheral structures of **G_3_OH**, whereas smaller DEN particles (such as **Co/G_4_OH-2** and **Co/G_4_OH-3**) are able to penetrate the fungal extracellular matrix more effectively.

It is significant that the synthesized nanocomposites exhibit activity comparable to or exceeding that of commercial antimycotics (nystatin, ketoconazole). For instance, the activity of **Co/G_4_OH-3** against *Candida albicans* (26.81 mm) surpasses that of ketoconazole (25.1 mm), while its activity against *Aspergillus niger* (26.99 mm) significantly exceeds that of nystatin (14.6 mm).

## 4. Conclusions

A **hyperbranched polyol process (HB-polyol process)** as a novel methodological approach for the one-step synthesis of colloidally stable cobalt metal–polymer nanocomposites (**Co/G_n_OH**) has been developed. It was demonstrated that bio-inspired hyperbranched polyester polyols of the third and fourth generations, **G_n_OH** (n = 3, 4), serve as effective reducing agents, macromolecular nanoreactors and stabilizers for low-dimensional (cluster) cobalt nanoparticles.

Unlike our previous work which was limited to demonstrating the reducing capacity of the **G_4_OH** polyol, this systematic comparative study of two generations of polyols (**G_3_OH** and **G_4_OH**) enables the formulation of fundamental physicochemical principles governing the HB-polyol process for the synthesis of metal–polymer nanocomposites.

Furthermore, fundamental differences have been established between the hyperbranched polyol process (HB-polyol process) and the classical linear polyol process (linear polyol process). Linear polyols, such as polyethylene glycol (PEG), exhibit a classical relationship: an increase in molecular weight enhances steric stabilization, leading to a reduction in the number of nuclei and, consequently, an increase in the size of the final particles [8,9,25]. In contrast, in the HB-polyol process, the polymer’s molecular weight is not an independent variable. It is inextricably linked to the degree of branching (DB) and the macromolecule’s topology. The increase in molecular weight upon transitioning from **G_3_OH** to **G_4_OH** correlates with an increase in the DB and conformational rigidity, driving a shift from surface coordination to intracavity encapsulation. This change does not merely slow down nucleation but fundamentally alters the ripening mechanism: the system shifts from Ostwald ripening to a “digestive ripening” mechanism, ultimately resulting in a decrease in particle size as the molecular weight of the dendrite-like polymer increases. A similar distinction is observed when analyzing the effect of the M^n+^/OH_polyol_ molar ratio on the nanocomposite size. While in linear polyols an increase in the precursor concentration leads to a decrease in the average nanoparticle diameter (classical LaMer nucleation mechanism) [19,23], in hyperbranched polyols, a similar size reduction is achieved through intracavity localization and a shift in the ripening mechanism toward digestive ripening, resulting from the steric constraints of the hyperbranched polyol core.

Based on a comprehensive analysis of the spectral and dispersion characteristics of the [CoCl_2_-G_n_OH] binary system, a **hierarchical concept of the HB-polyol process** has been established. According to this concept, the morphology and functional properties of **Co/G_n_OH** nanocomposites are determined by the interdependent influence of three key parameters: **(i)** the spatial architecture of the hyperbranched polyol, **(ii)** the type of metal nanophase localization and **(iii)** the chemical composition of the polymer surface after in situ oxidation. These parameters exert a sequential cascading effect on three stages of nanoparticle formation: the pre-organization of metal ions within the polyol matrix, nucleation, and polyol oxidation, followed by nanophase stabilization.

At the pre-organization stage, the **spatial architecture of the polymer**, specifically the degree of branching (DB), serves as the primary factor governing the binding nature of metal ions. The open architecture of **G_3_OH** (DB = 0.37) ensures surface-type localization via intermolecular coordination of Co^2+^ ions with terminal OH groups of the polyol. An increase in the DB and, consequently, in the conformational rigidity of the macromolecule with increasing generation, promotes intracavity localization via intramolecular coordination of Co^2+^ ions with linear OH groups within the pre-organized “polar pockets” of **G_4_OH** (DB = 0.48).

The established type of metal ion localization dictates the subsequent nucleation and maturation kinetics of the metallic nanophase. For **G_3_OH**, where Co^2+^ ions are pre-organized within the functional shell of the dendritic polyol, the direct Ostwald ripening mechanism prevails, characterized by a progressive increase in the hydrodynamic diameter (d_h_) from 74 to 103 nm and increasing polydispersity with rising metal loading. In **G_4_OH**, which encapsulates cobalt ions, a transition to the digestive ripening mechanism occurs under the nanoconfinement conditions of the polyol’s hydrophobic core. This shift in the ripening mechanism is characterized by a monotonic decrease in d_h_ and monodispersity of particles with increasing cobalt nanophase content. Importantly, the final formation of the colloidally stable nanophase is completed within 5–7 days, as verified by the synchronization of the surface plasmon resonance (SPR) maximum plateauing (λ_SPR_ = 273–279 nm) with the evolution of the NTA parameter profiles (hydrodynamic diameter d_h_ and particle concentration C_NP_).

In turn, the **type of cobalt nanophase localization** within the hyperbranched polyol nanoreactor determines the depth of oxidation and the chemical composition of the polymer shell, as well as the subsequent mechanism of cobalt nanophase stabilization. Using in situ diffuse reflectance FT-IR spectroscopy with Gaussian deconvolution, it was proven that surface-localized cobalt clusters in **G_3_OH** act as heterogeneous catalysts, promoting the deep oxidation of terminal alcohol groups to carboxyl groups, as evidenced by the increase in the C=O_acid_ band area from 10.69 to 25.12. Conversely, the encapsulation of the cobalt nanophase within **G_4_OH** shields the nascent nanoclusters from the external environment, blocking the catalytic cascade and limiting the oxidation of peripheral OH groups exclusively to aldehyde groups (the C=O_aldehyde_ band area remains stable at ~0.33–0.35 throughout the heating process).

Ultimately, the **chemical composition of the hyperbranched polymer** surface after in situ oxidation (carboxyl vs. aldehyde groups) serves as the final trigger governing supramolecular assembly. It dictates not only the stabilization mode (chelation vs. coordination) but also the shape (layered aggregates vs. spherical vesicles) and the dimensional characteristics of the colloidal metal–polymer **Co/G_n_OH** nanoparticles. The carboxylated shell of oxidized **G_3_OH** acquires a chelating ability which induces strong intermolecular cross-linking, leading to the formation of polydisperse “flake-like” layered structures with a wide range of particle sizes (from 35 ± 5 to 4 ± 1 nm) and an increase in the hydrodynamic diameter due to aggregation. The aldehyde surface of oxidized **G_4_OH** provides only weak monodentate coordination incapable of cross-linking macromolecules. Consequently, the nanocomposites are represented exclusively by monodisperse spherical nanovesicles, whose core size predictably decreases from 50 ± 7 to 13 ± 3 nm with increasing metal loading while the hydrodynamic diameter monotonically decreases due to the compaction effect.

Thus, the proposed hierarchical concept demonstrates that the initially defined hyperbranched polymer architecture serves as a programmable template for the HB-polyol process. Selecting the generation of the polyol matrix allows for targeted control over ionic pre-organization, directing the nucleation mechanism and fine-tuning the chemical composition of the stabilizing shell. The research results present a new mechanism for controlling morphology for the rational design of **Co/G_n_OH** nanocomposites with tailored morphology, magnetic response and biological activity. This positions the HB-polyol process as a powerful tool for creating “smart” non-toxic nanomaterials for advanced biomedical applications.

## Figures and Tables

**Table 1:** The sorption parameters obtained by UV-Vis and dispersion characteristics obtained by the NTA method for [Co^2+^-G_n_OH] systems.

System	K_L_, mL × mM^−1^	A_∞_, mM × g ^−1^	d_h_^mean^, nm	C_NP_ 10^8^, Particles × mL^−1^	PDD
G_3_OH			106 ± 3	1.85 ± 0.28	0.74 ± 0.03
G_4_OH			134 ± 2	1.00 ± 0.76	0.76 ± 0.04
[Co^2+^–G_3_OH]	3.03 ± 0.15	2.79 ± 0.14	282 ± 14	0.08 ± 0.01	0.58 ± 0.08
[Co^2+^–G_4_OH]	3.51 ± 0.18	3.20 ± 0.16	143 ± 6	1.55 ± 0.17	0.81 ± 0.08

**Table 2:** Particle characteristics obtained by NTA (mean hydrodynamic diameter—d_h_^mean^, particle concentration—C_NP_, PDD) and by TEM methods for **Co/G_n_OH** nanocomposites.

Sample	d_h_^mean^, nm	C_NP_ × 10^8^, Particles × mL^−1^	Dispersity	PDD	d_TEM_, nm
Co/G_3_OH-1	74 ± 6	1.44 ± 0.03	poly	0.65 ± 0.03	35 ± 5
Co/G_3_OH-2	79 ± 3	2.66 ± 0.02	mono	0.82 ± 0.04	31 ± 5
Co/G_3_OH-3	103 ± 6	3.1 ± 0.4	poly	0.68 ± 0.03	4 ± 1
Co/G_4_OH-1	149 ± 5	4.4 ± 0.2	mono	0.87 ± 0.03	50 ± 7
Co/G_4_OH-2	66 ± 1	4.9 ± 0.2	mono	0.85 ± 0.03	28 ± 4
Co/G_4_OH-3	53 ± 2	5.7 ± 0.2	mono	0.87 ± 0.03	13 ± 3

**Table 3:** The evaluated chi_0_ parameter values from the room-temperature data.

Sample	chi_0_, emu g^−1^ Oe^−1^
Co/G_3_OH-3	−6.65·10^−7^
Co/G_3_OH-2	−6.02·10^−7^

**Table 4:** The value of cores diameters and total magnetic moment evaluated by fitting.

Sample	The Diameter of a Magnetic Core, nm	The Total Average Magnetic Moment of a Core, μ_B_
Co/G_3_OH-3	1.4	0.268
Co/G_3_OH-2	7.2	0.002

**Table 5:** The hemolysis coefficient value corresponding to the destruction of 50% of red blood cells is HC_50_.

Sample	HC_50_, mg × mL^−1^	Sample	HC_50_, mg × mL^−1^
CoCl_2_	2.82		
G_3_OH	17.91	**G_4_OH**	16.94
Co/G_3_OH-1	2.85	**Co/G_4_OH-1**	8.89
Co/G_3_OH-2	1.98	**Co/G_4_OH-2**	7.83
Co/G_3_OH-3	1.41	**Co/G_4_OH-3**	1.39

**Table 6:** Disk diffusion test data (diameter of lysis zones, mm) from tests of antifungal activity on pathogenic cultures (standard deviation SD < 5%).

Diameter of Lysis Zones (mm)					
Sample	*Candida albicans RCPF*	*Candida albicans ATCC 10201*	*Cryptococcus laurentii*	*Aspergillus fumigatus F1119*	*Aspergillus niger F753*
G_3_OH	0	0	0	0	0
Co/G_3_OH-1	12.99	21.17	13.78	0	12.6
Co/G_3_OH-2	11.3	19.76	7.29	0	19.4
Co/G_3_OH-3	12.95	32.87	15.47	0	22.8
G_4_OH	0	0	0	0	0
Co/G_4_OH-1	6.43	0	0	0	24.3
Co/G_4_OH-2	15.06	11.64	14.79	10.15	9.08
Co/G_4_OH-3	26.81	16.13	23.2	9.82	6.99
Nystatin	15.5	14.6	23.2	11.7	14.6
Ketoconazole	25.1	31.8	16.2	0	10.7

## Data Availability

The original contributions presented in this study are included in the article/Supplementary Material. Further inquiries can be directed to the corresponding authors.

## Supplementary Materials

The following supporting information can be downloaded at https://www.mdpi.com/article/10.3390/nano16150960/s1: Figure S1. Band–resolved UV-Vis spectra of CoCl_2_ in DMSO; Figure S2. Distribution by size and number of particles in aqueous solution of G_n_OH [Co^2+^-G_3_OH] and [Co^2+^-G_4_OH], isotherms of sorption of Co^2+^ ions by G_3_OH and G_4_OH matrix in DMSO solution; Figure S3. ^1^H NMR spectra of G_3_OH and G_4_OH in (CD_3_)_2_SO; Figure S4. Appearance of the Co/G_n_OH reaction systems after completion of the synthesis; Figure S5. Evolution of electronic absorption spectra of dispersions of individual polymer-stabilized Co/G_3_OH-1; Figure S6. NTA Data of nanocomposite dispersions of Co/G_3_OH-1, Co/G_3_OH-2, Co/G_3_OH-3, Co/G_4_OH-1, Co/G_4_OH-2, Co/G_4_OH-3; Figure S7. FT-IR spectra of diffuse reflectance of [CoCl_2_-G_3_OH] and [CoCl_2_-G_4_OH] mixture in situ when heated; Figure S8. Profiles of intensity of characteristic bands in the IR diffuse reflectance spectra of the [CoCl_2_-G_3_OH] and [CoCl_2_-G_4_OH] mixture depending on the heating; Figure S9. FT-IR spectra of diffuse reflectance of G_4_OH mixture in situ when heated; Figure S10. FT-IR spectra and the region of ester bonds with deconvolution for the initial and obtained compounds.; Figure S11. TEM images, particle size distribution and electron diffraction of Co/G_3_OH and Co/G_4_OH; Figure S12. NTA data of nanocomposite dispersions Co/G_3_OH (A) and Co/G_4_OH (B) in the DMSO; Figure S13. TG-DTG curves of Co/G_3_OH (A) and Co/G_4_OH (B) nanocomposites; Figure S14. Zone of growth inhibition in assessing the sensitivity of *Candida albicans RCPF*, *Candida albicans ATCC 10201*, *Cryptococcus laurentii, Aspergillus niger F753* and *Aspergillus fumigatus F1119* to Co/G_3_OH; Figure S15. Zone of growth inhibition in assessing the sensitivity of *Candida albicans RCPF*, *Candida albicans ATCC 10201*, *Cryptococcus laurentii*, *Aspergillus niger F753* and *Aspergillus fumigatus F1119* (F) to Co/G_4_OH.
