# Supplementary material for: Hyperbranched Polyol Process for the Synthesis of Multifunctional Cobalt Nanocomposites: Interplay of Polymer Architecture, Metal Localization and Material Properties
Source: Nanomaterials (Basel). 2026 Aug 4;16(15):960. doi: 10.3390/nano16150960 (PMC13468392; doi:10.3390/nano16150960)
Supplement: Supplementary file 1 [file nanomaterials-16-00960-s001.zip › nanomaterials-4471317-supplementary.pdf]

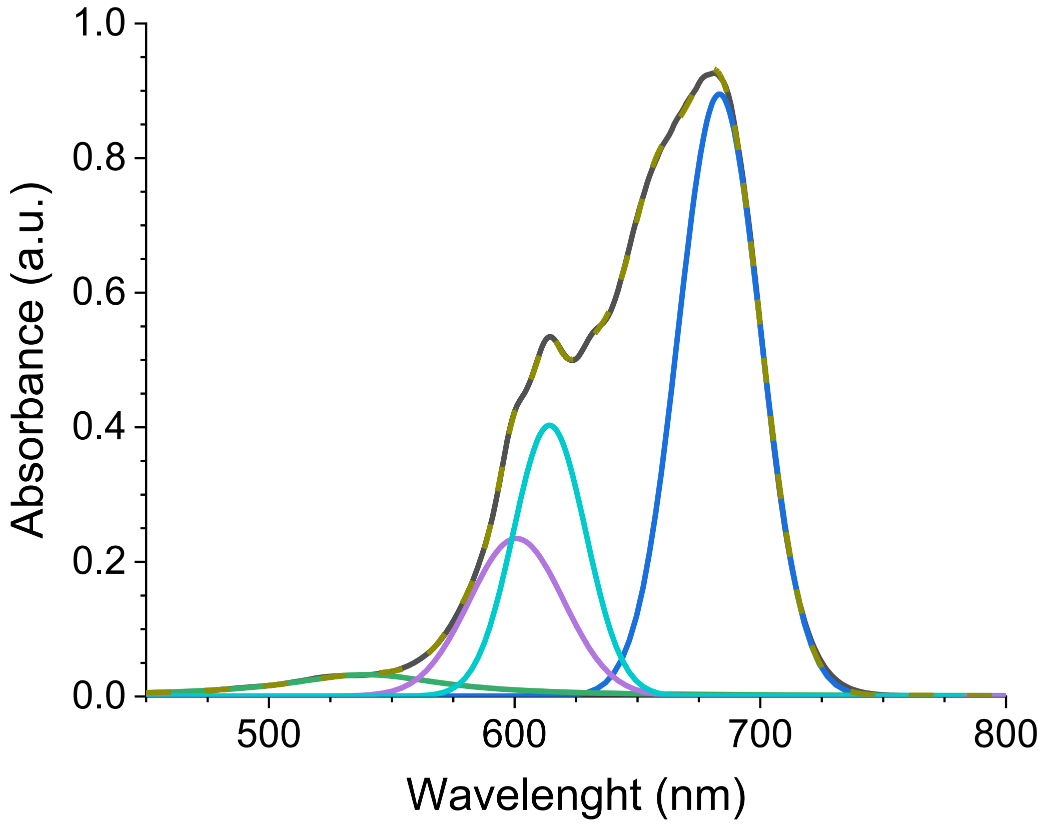

Figure S1. Band – resolved UV-Vis spectra of $CoCl_2$ in DMSO (C_{CoCl2}= 0.05 mol·l^{-1}) (R^2 = 99.6, χ^2 = 7.23·10^{-6}).

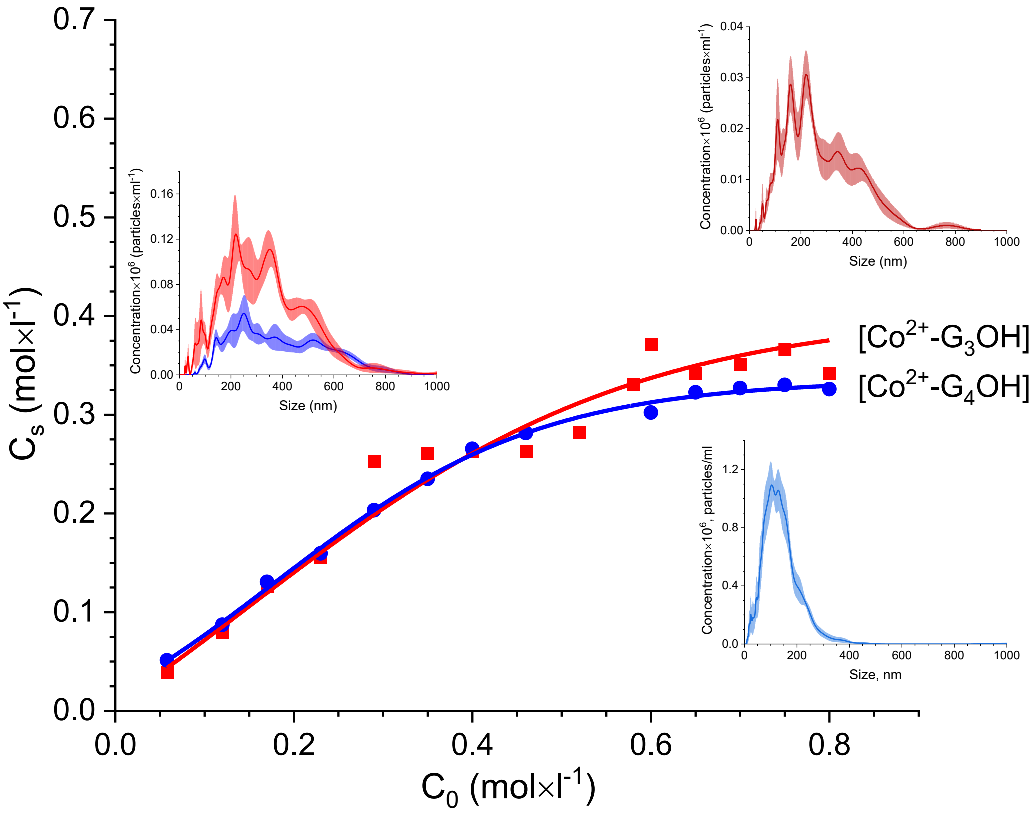

Figure S2. Distribution by size and number of particles in aqueous solution of GnOH [Co^{2+} – G_3OH] and [Co^{2+} – G_4OH], isotherms of sorption of Co^{2+} ions by G_3OH and G_4OH matrix in DMSO solution (C_{GnOH} = 0.125 g × ml^{-1}, C_{Co2+} = 0.05-0.8 M).

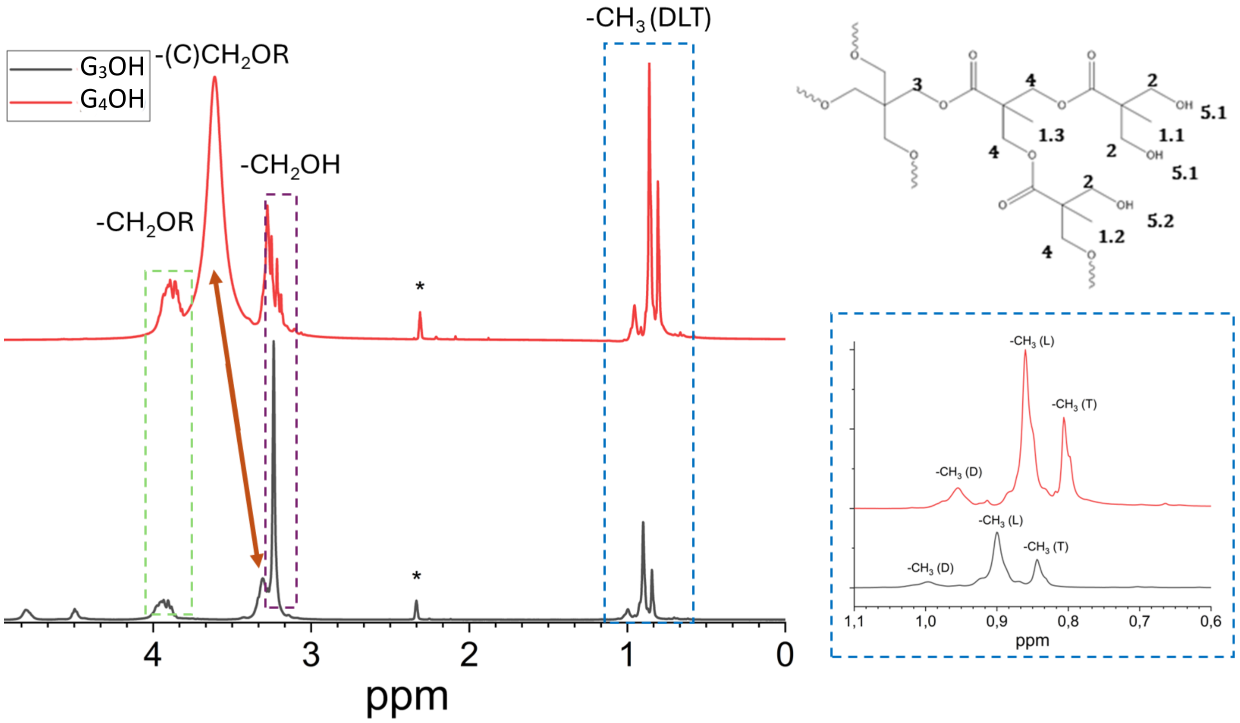

Figure S3. ^{1}H NMR spectra of G_3OH and G_4OH in $(CD_3)_2SO$.

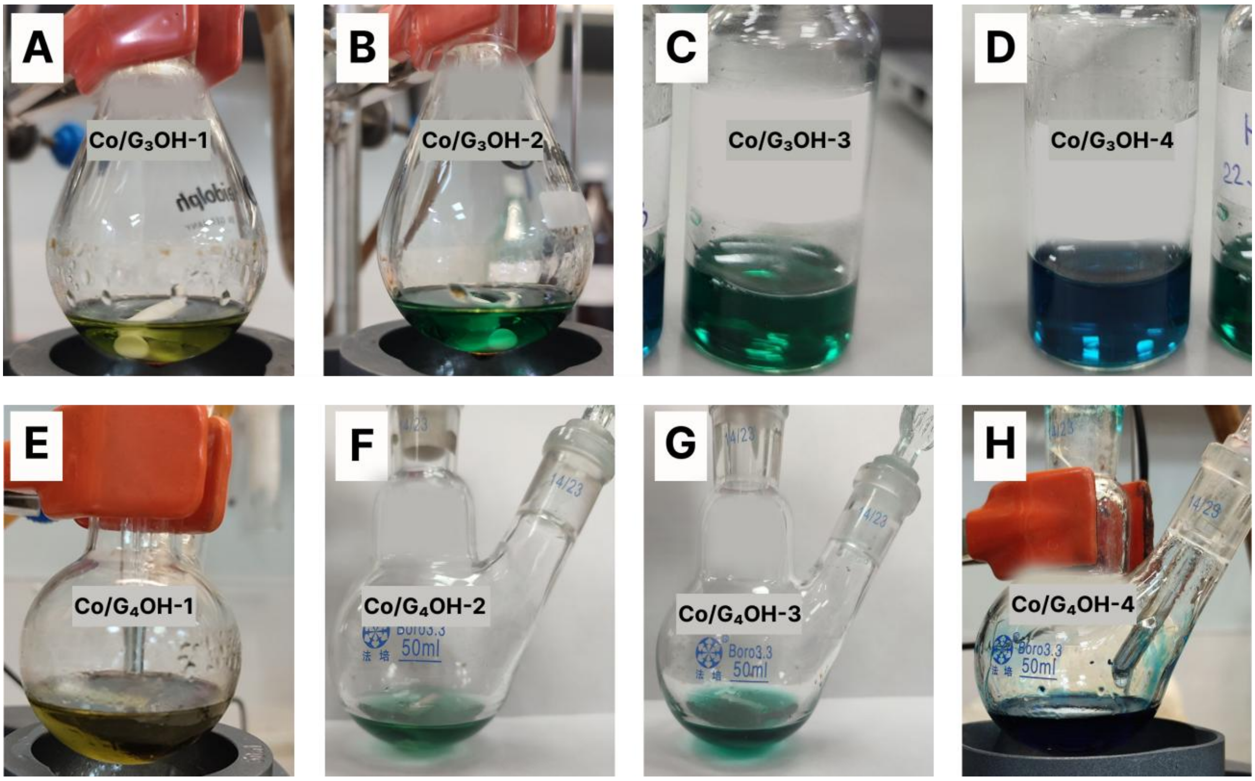

Figure S4. Appearance of the Co/G$_n$OH reaction systems after completion of the synthesis: A-Co/G$_3$OH-1, B- Co/G$_3$OH-2, C- Co/G$_3$OH-3, D - Co/G$_3$OH-4, E- Co/G$_4$OH-1, F- Co/G$_4$OH-2, G- Co/G$_4$OH-3, H- Co/G$_4$OH-4.

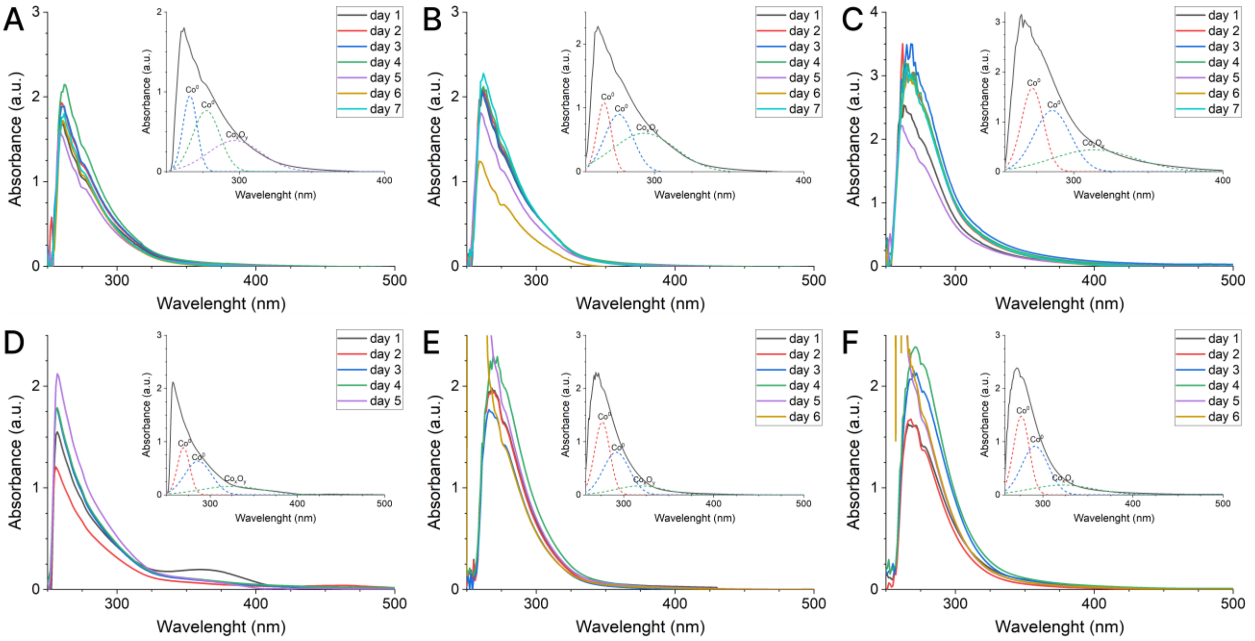

Figure S5. Evolution of electronic absorption spectra of dispersions of individual polymer-stabilized Co/G_3OH-1 (A) ($R^2 = 99.6$, $\chi^2 = 7.41\cdot10^{-5}$), Co/G_3OH-2 (B) ($R^2 = 99.8$, $\chi^2 = 5.41\cdot10^{-4}$), Co/G_3OH-3 (C) ($R^2 = 99.7$, $\chi^2 = 4.65\cdot10^{-5}$), Co/G_4OH-1 (D) ($R^2 = 99.8$, $\chi^2 = 5.42\cdot10^{-4}$), Co/G_4OH-2 (E) ($R^2 = 99.7$, $\chi^2 = 9.72\cdot10^{-4}$), Co/G_4OH-3 (F) ($R^2 = 99.7$, $\chi^2 = 1.37\cdot10^{-5}$) nanoparticles in water ($C_{Co/GnOH}$ = 2 mg/ml) and interpolation of absorption band from Gauss distribution function.

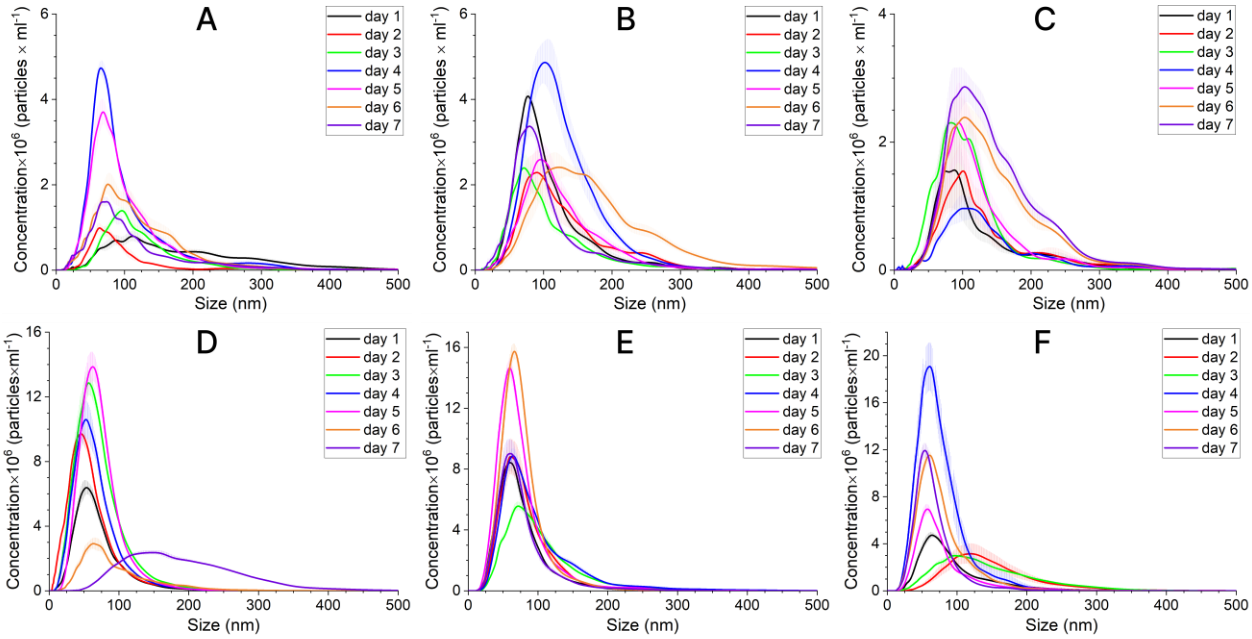

Figure S6. NTA Data of nanocomposite dispersions of Co/G$_3$OH-1 (A), Co/G$_3$OH-2 (B), Co/G$_3$OH-3 (C), Co/G$_4$OH-1 (D), Co/G$_4$OH-2 (E), Co/G$_4$OH-3 (F) ($C_{Co/GnOH}$ = 2 mg/ml) in the DMSO.

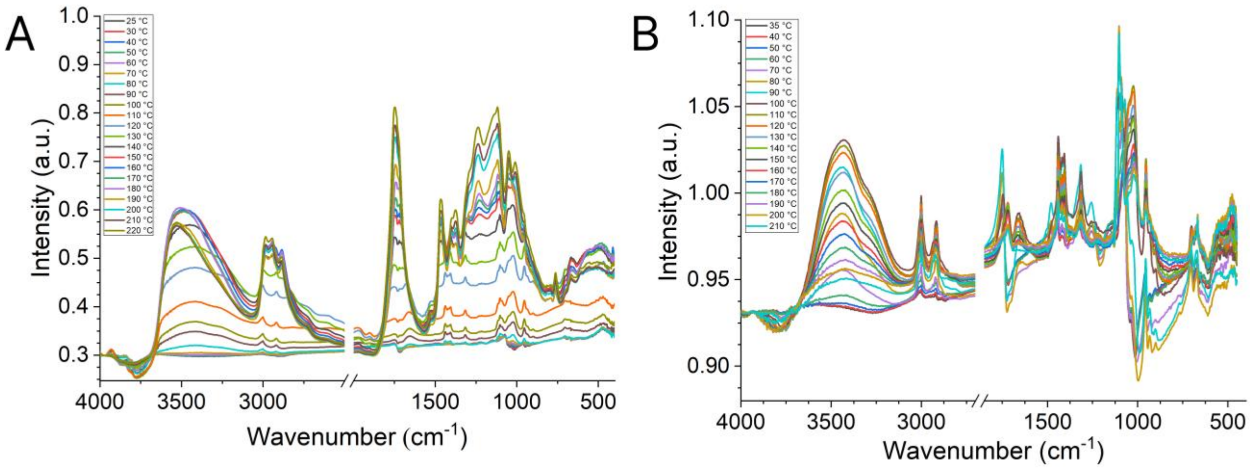

Figure S7. FT-IR spectra of diffuse reflectance of [$CoCl_2$-G_3OH] (A) and [$CoCl_2$-G_4OH] (B) mixture in situ when heated (T=35-210 °C, C_{G3OH} = 0.629 g × ml^{-1}, C_{Co2+} = 0.00258 g × ml^{-1}).

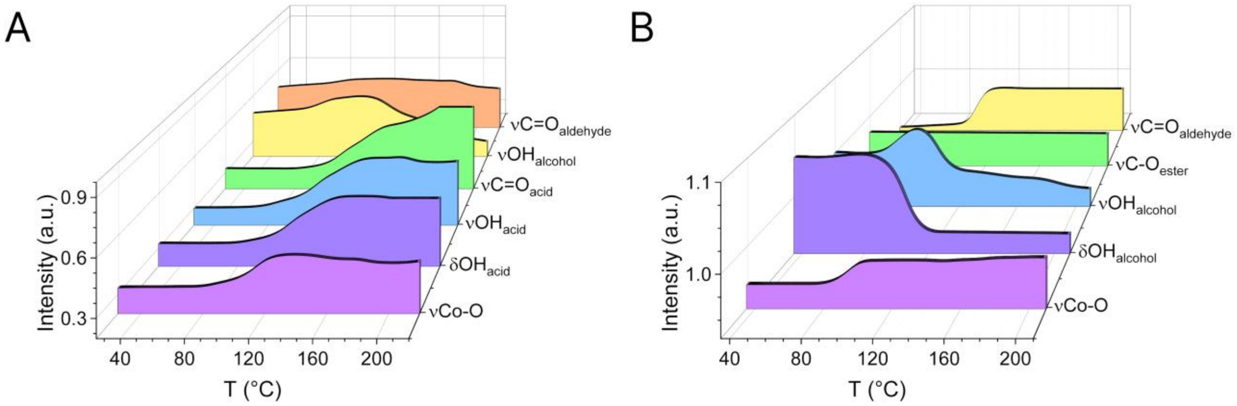

Figure S8. Profiles of intensity of characteristic bands in the IR diffuse reflectance spectra of the [$CoCl_2$-G_3OH] (A) and [$CoCl_2$-G_4OH] (B) mixture depending on the heating (T=35-210 °C, C_{G4OH} = 0.629 g × ml^{-1}, C_{Co2+} = 0.00258 g × ml^{-1}).

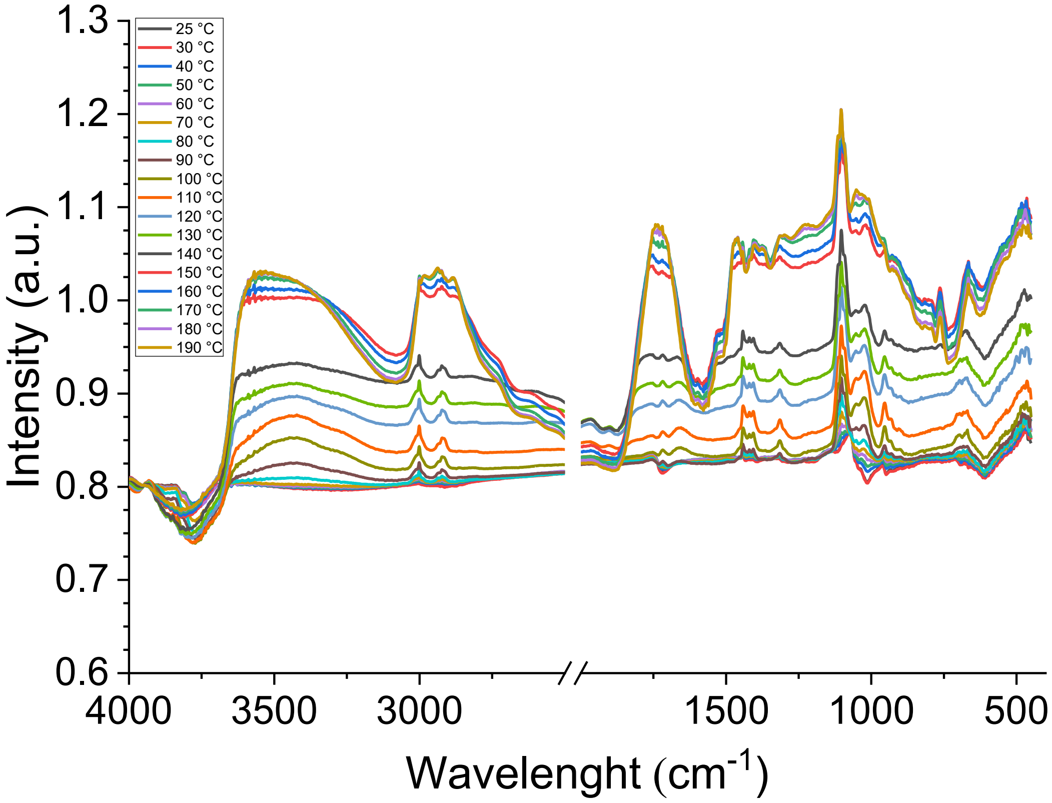

Figure S9. FT-IR spectra of diffuse reflectance of G_4OH mixture *in situ* when heated (T=35-210 °C, C_{G3OH} = 0.629 g × ml^{-1}).

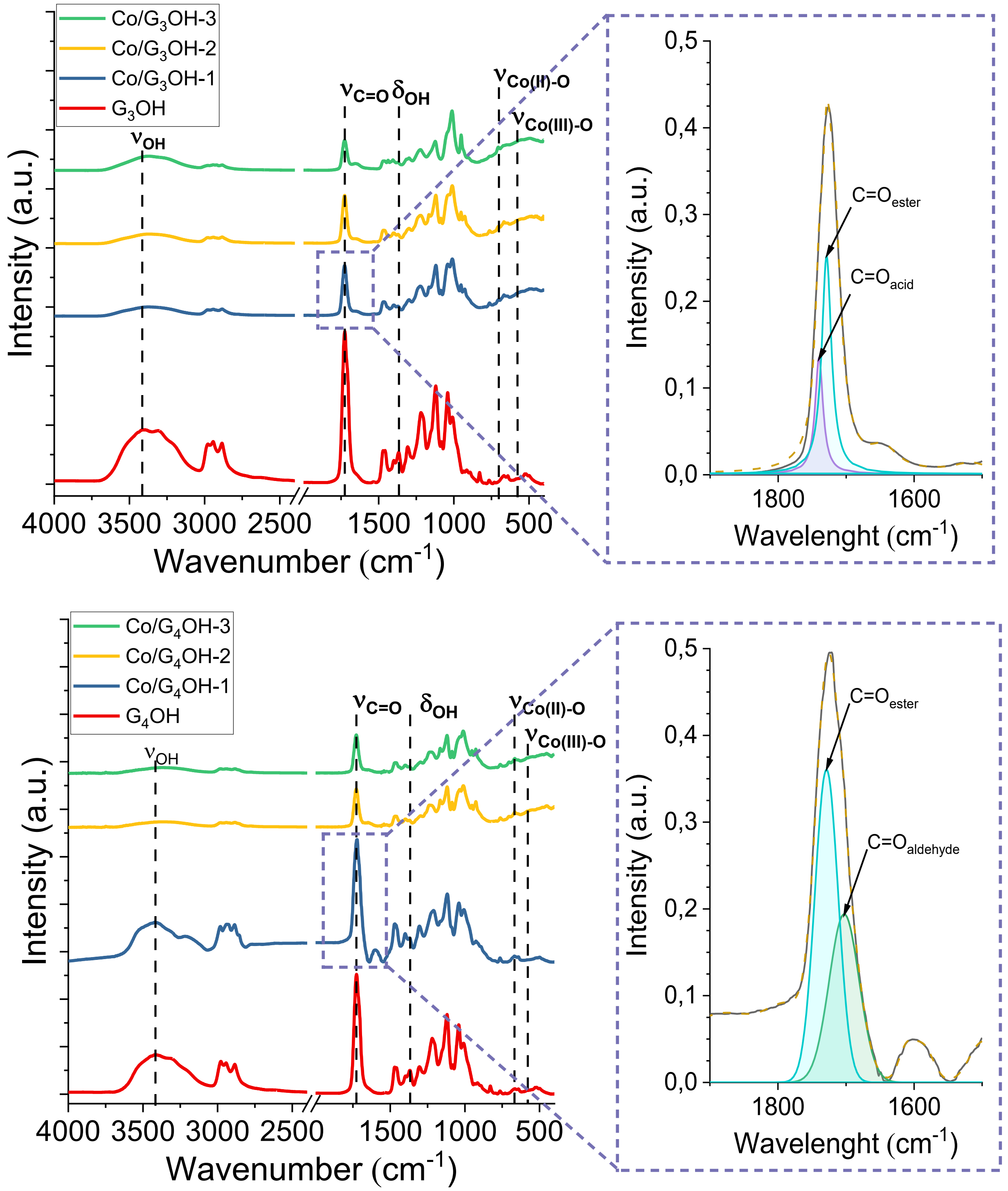

Figure S10. FT-IR spectra and the region of ester bonds with deconvolution for the initial and obtained compounds.

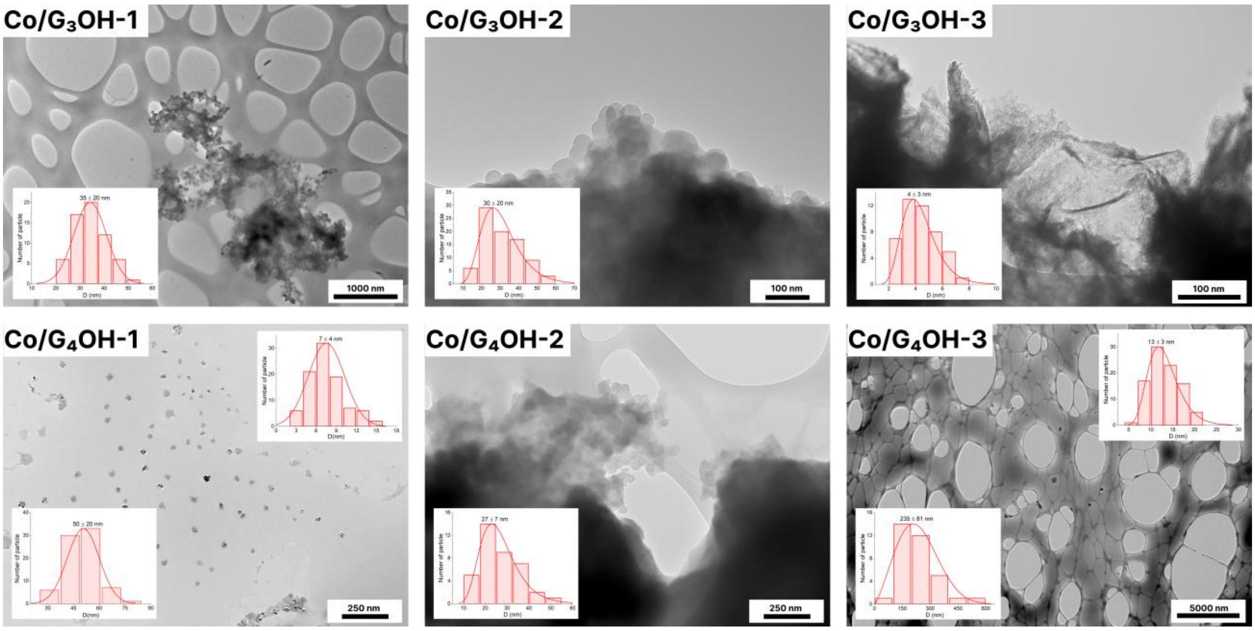

Figure S11. TEM images, particle size distribution and electron diffraction of Co/G_3OH and Co/G_4OH.

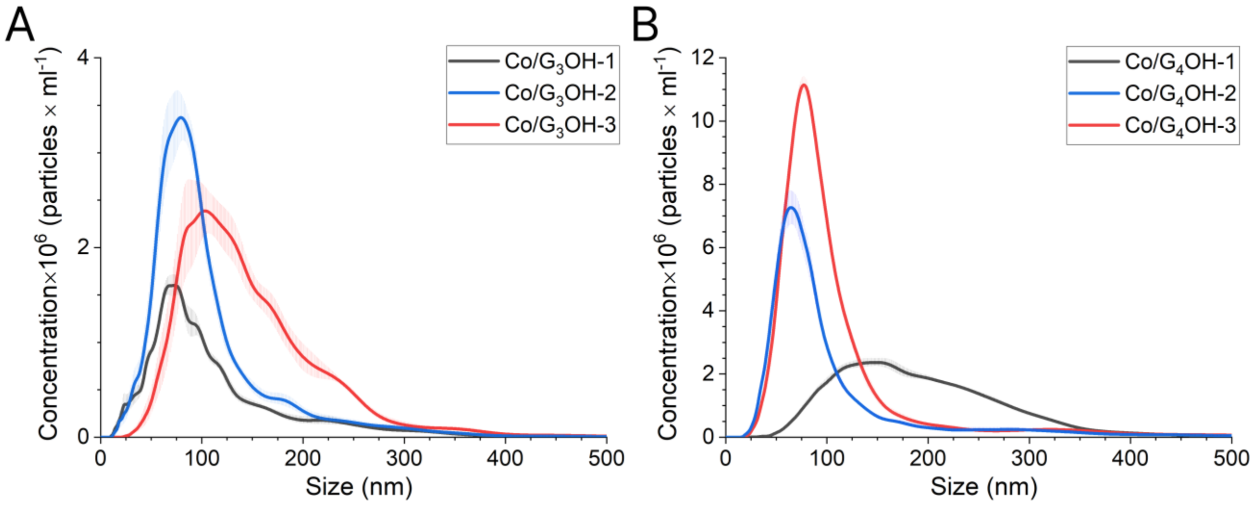

Figure S12. NTA data of nanocomposite dispersions Co/G_3OH (A) and Co/G_4OH (B) in the DMSO.

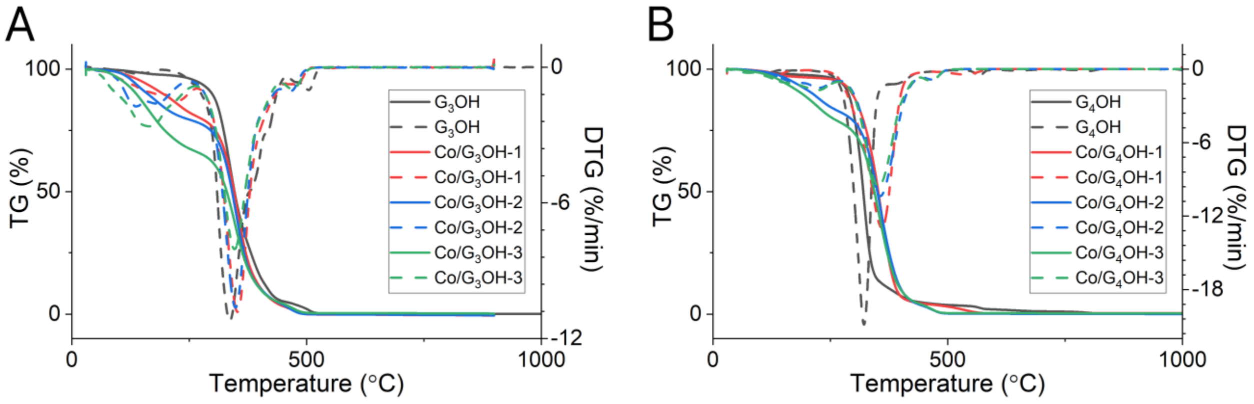

Figure S13. TG-DTG curves of Co/G_3OH (A) and Co/G_4OH (B) nanocomposites.

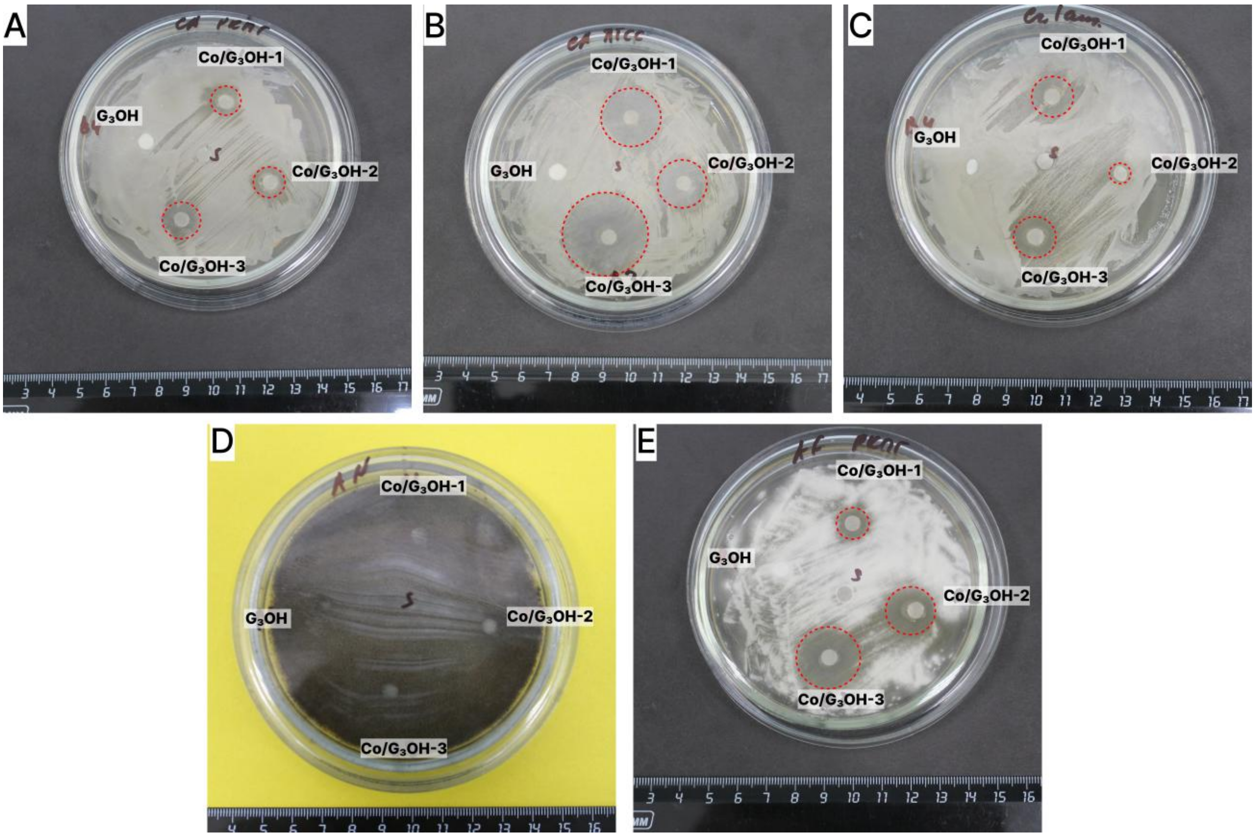

Figure S14. Zone of growth inhibition in assessing the sensitivity of *Candida albicans RCPF* (A), *Candida albicans ATCC 10201* (B), *Cryptococcus laurentii* (C), *Aspergillus niger F753* (D) and *Aspergillus fumigatus F1119* (F) to Co/G_3OH.

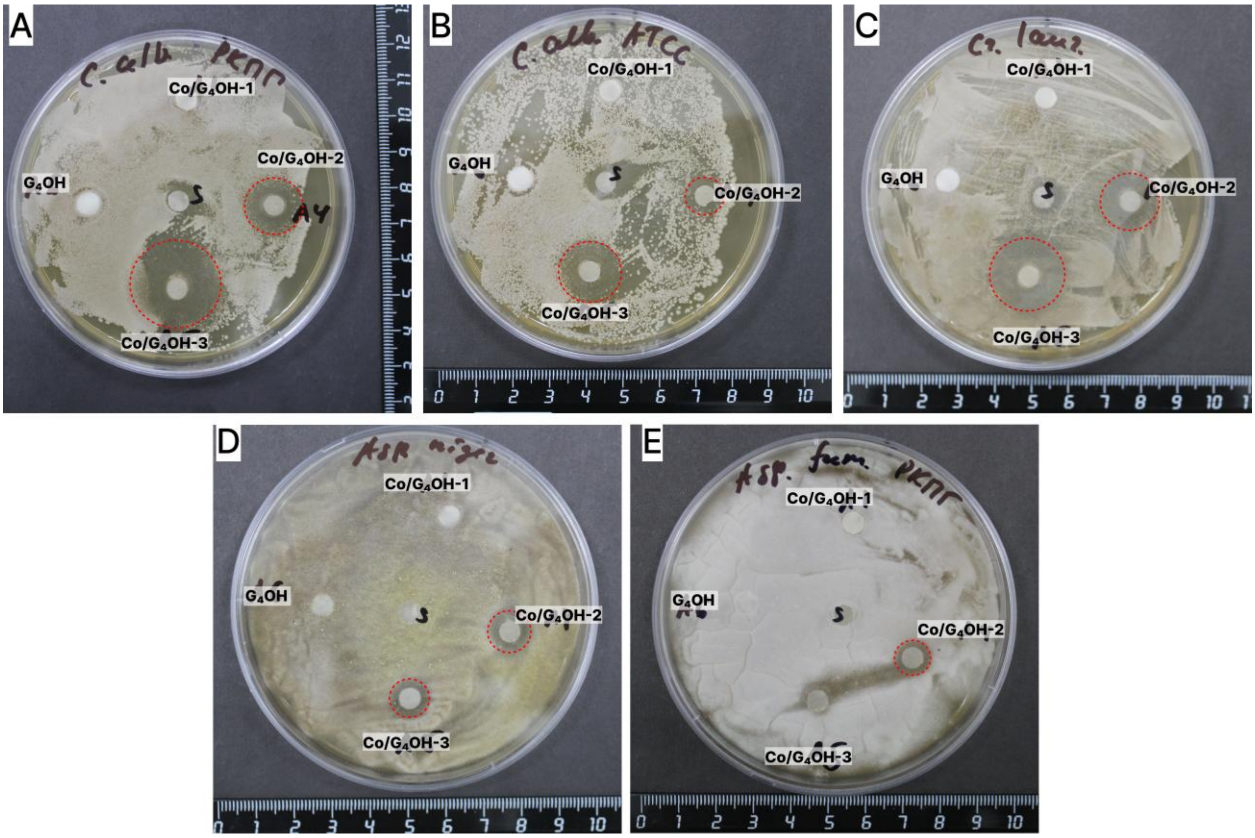

Figure S15. Zone of growth inhibition in assessing the sensitivity of *Candida albicans RCPF* (A), *Candida albicans ATCC 10201* (B), *Cryptococcus laurentii* (C), *Aspergillus niger F753* (D) and *Aspergillus fumigatus F1119* (F) to Co/G_4OH.
